# Supplementary figures and images for: SUITOR: Selecting the number of mutational signatures through cross-validation
Source: PLoS Comput Biol. 2022 Apr 4;18(4):e1009309. doi: 10.1371/journal.pcbi.1009309 (PMC9009674; doi:10.1371/journal.pcbi.1009309)

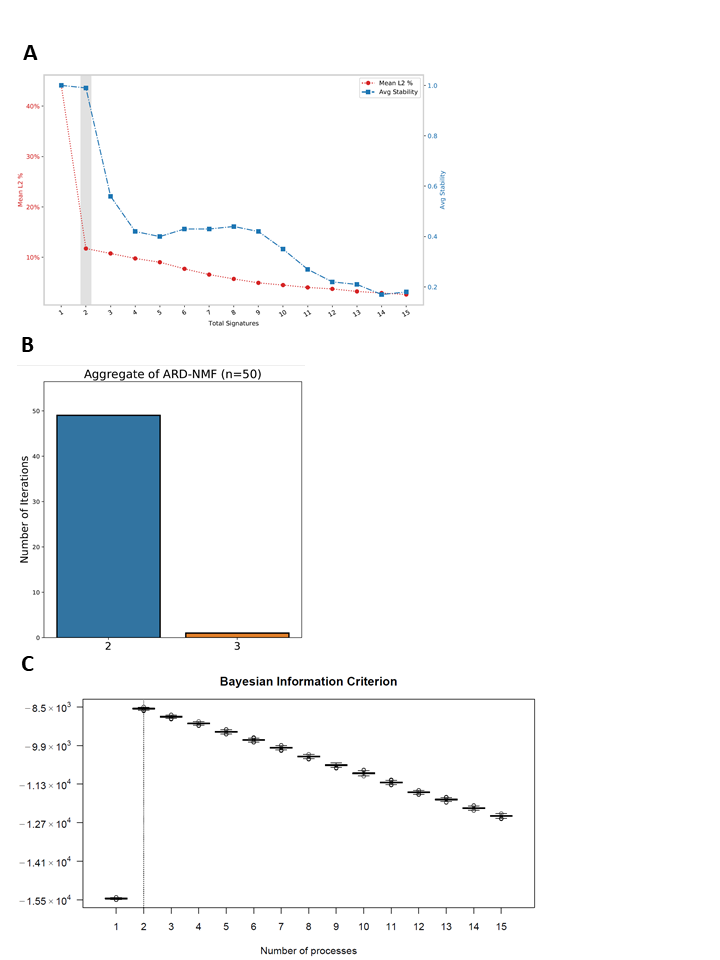

Supplement: S1 Fig — Each plot shows how SigProfilerExtractor (A), SignatureAnalyzer (B) and signeR (C) select the optimal number of signatures respectively. (TIF) [file pcbi.1009309.s001.tif]

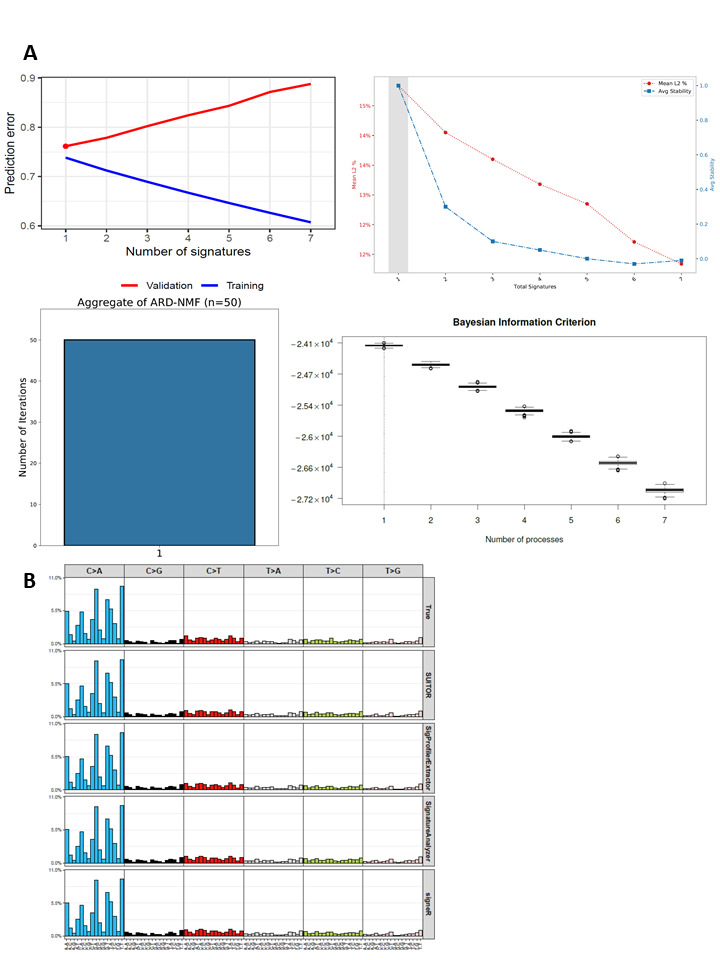

Supplement: S2 Fig — A) The plots of criteria to select the optimal number of signatures by SUITOR, SigProfilerExtractor, signeR and SignatureAnalyzer (in clockwise order). B) The profiles of signatures discovered by each method. (TIF) [file pcbi.1009309.s002.tif]

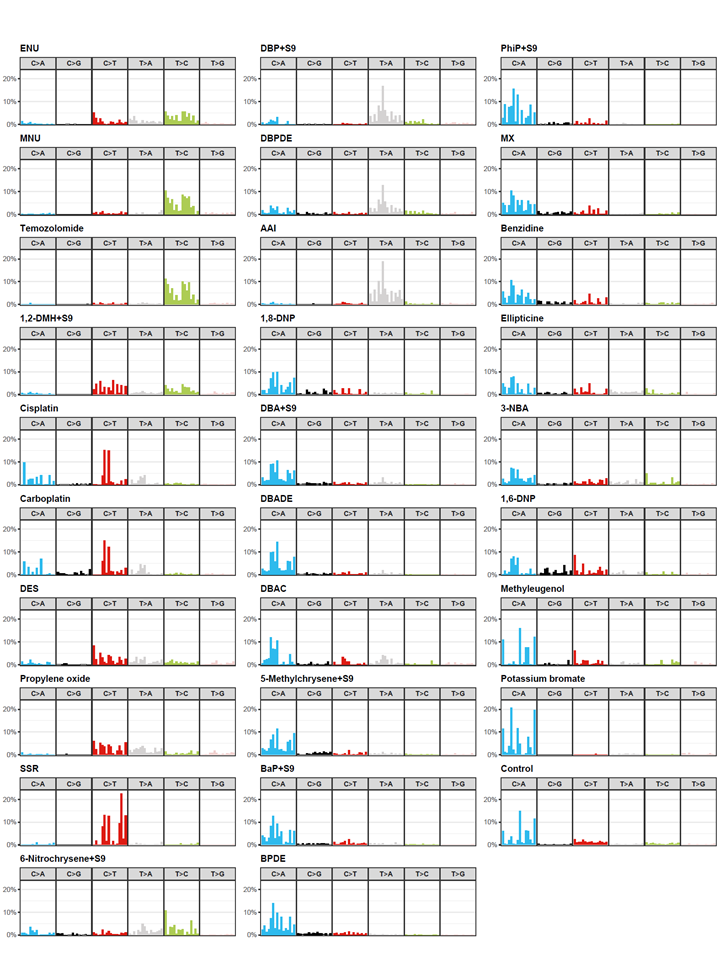

Supplement: S3 Fig — The signatures include 28 mutagen-induced signatures and a background signature existing in control samples and all mutagen treated samples. ENU: N-ethyl-N-nitrosourea; DBP+S9: dibenzo[a,l]pyrene mixed with S9 rodent liver-derived metabolic enzyme; PhiP+S9: 2-amino-1-methyl-6-phenylimidazo[4,5-b]pyridine mixed with S9 rodent liver-derived metabolic enzyme; MNU: N-methyl-N-nitrosourea; DBPDE: dibenzo[a,l]pyrene diol-epoxide; MX: 3-chloro-4-(dichloromethyl)-5-hydroxy- 2(5H)-furanone; AAI: aristolochic acid I; 1,2-DMH+S9: 1,2-dimethylhydrazine mixed with S9 rodent liver-derived metabolic enzyme; 1,8-DNP: 1,8-Dinitropyrene; DBA+S9: dibenz[a,h]anthracene mixed with S9 rodent liver-derived metabolic enzyme; 3-NBA: 3-nitrobenzanthrone; DBADE: dibenz[a,h]anthracene diol-epoxide; 1,6-DNP: 1,6-Dinitropyrene; DES: diethyl sulfate; DBAC: dibenz[a,j]acridine; 5-Methylchrysene+S9: 5-Methylchrysene mixed with S9 rodent liver-derived metabolic enzyme; SSR: simulated solar radiation; BaP+S9: benzo[a]pyrene mixed with S9 rodent liver-derived metabolic enzyme; 6-Nitrochrysene+S9: 6-Nitrochrysene mixed with S9 rodent liver-derived metabolic enzyme; BPDE: benzo[a]pyrene-7,8-dihydrodiol-9,10-epoxide. (TIF) [file pcbi.1009309.s003.tif]

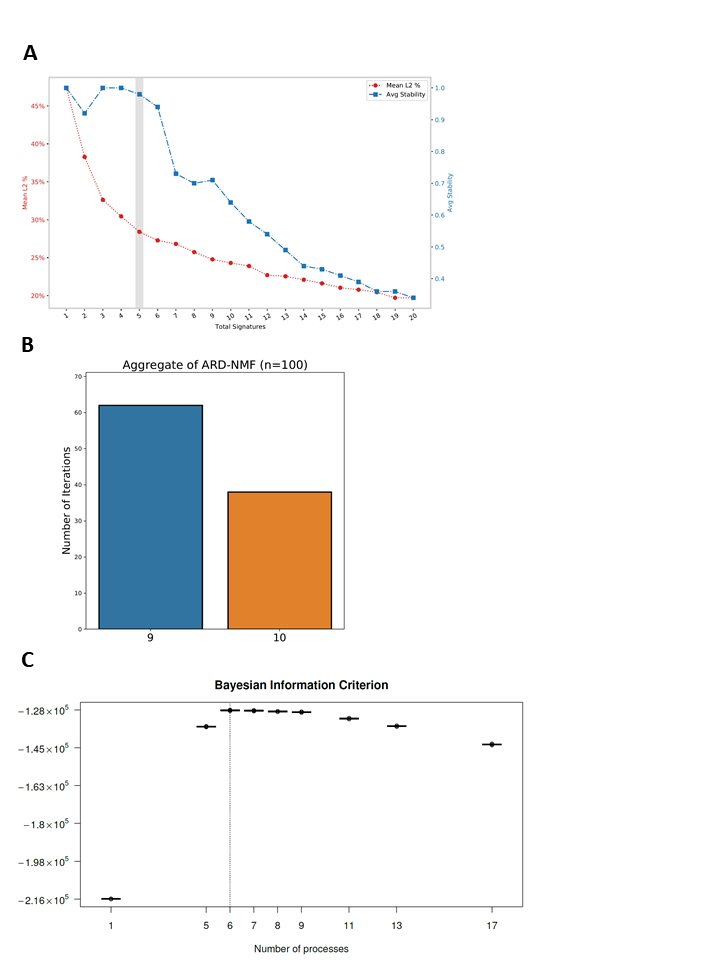

Supplement: S4 Fig — The number of signatures selected from the in vitro study of environmental or therapeutic mutagens by A) SigProfilerExtractor, B) SignatureAnalyzer and C) signeR. The numbers of detected signatures include one background signature and additional mutagen-induced mutational signatures. (TIF) [file pcbi.1009309.s004.tif]

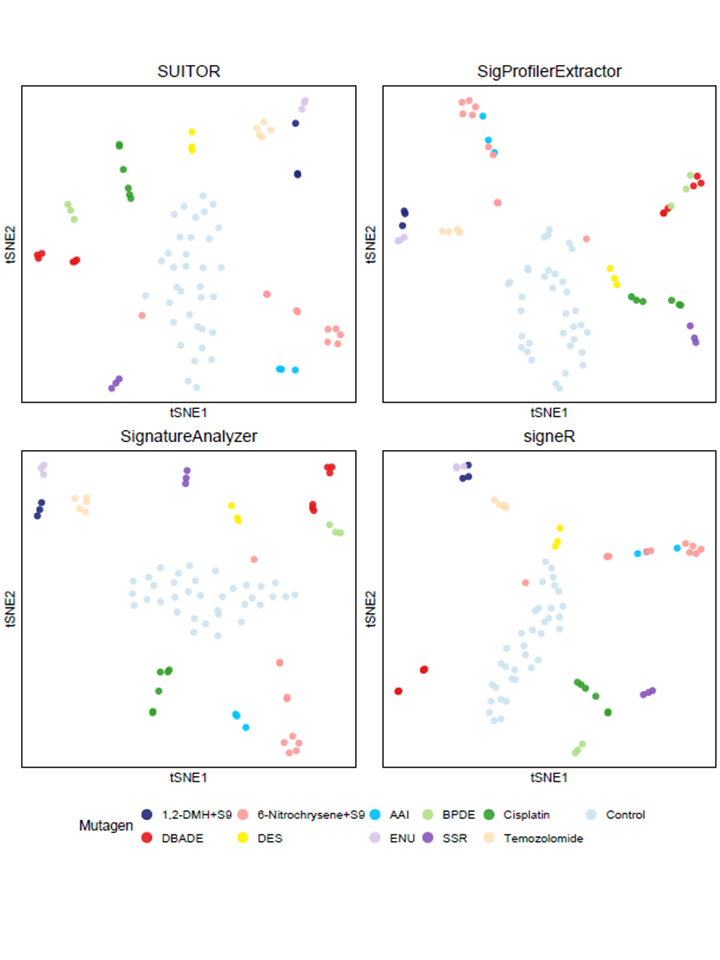

Supplement: S5 Fig — Each dot represents a subclone, colored by the mutagen treatment. (TIF) [file pcbi.1009309.s005.tif]

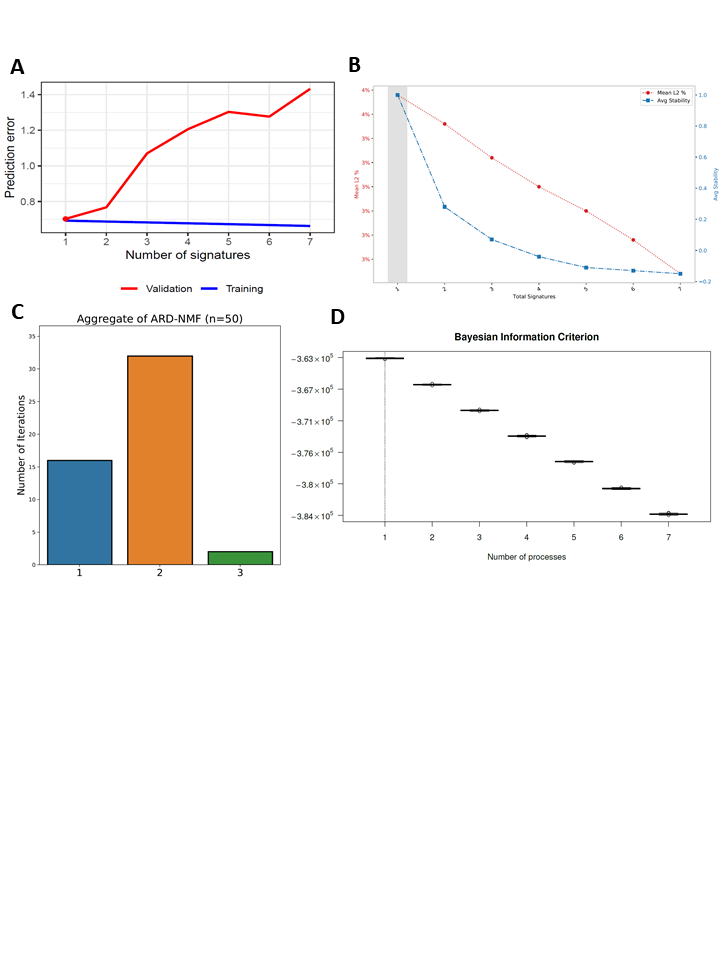

Supplement: S6 Fig — The number of signatures selected for in silico simulation studies with one signature by SUITOR (A), SigProfilerExtractor (B), SignatureAnalyzer (C) and signeR (D) for one replicate as an illustration. (TIF) [file pcbi.1009309.s006.tif]

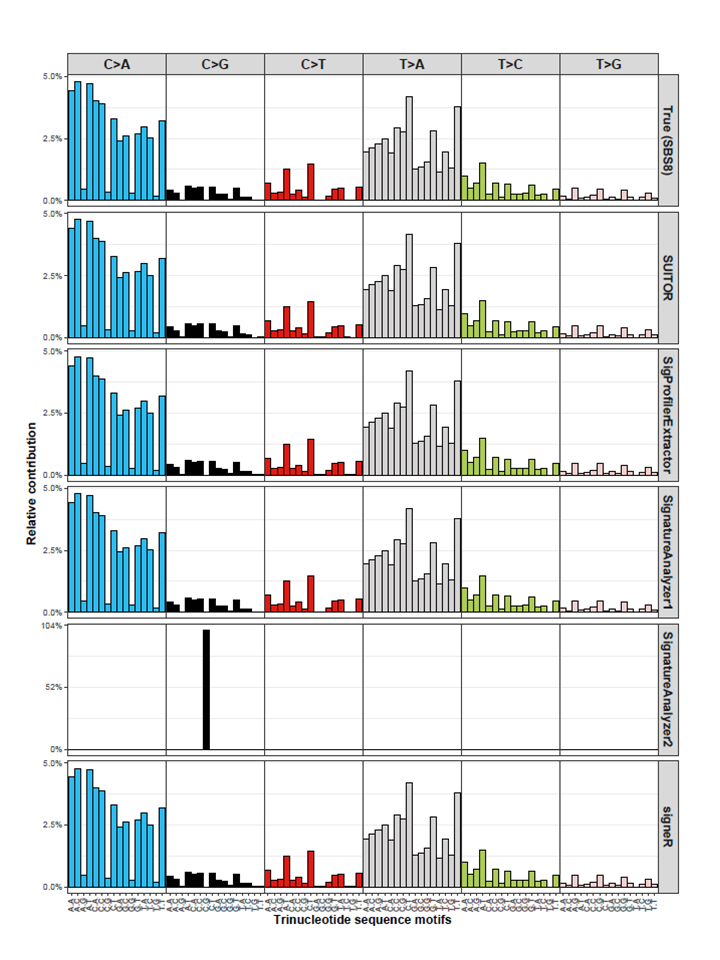

Supplement: S7 Fig — (TIF) [file pcbi.1009309.s007.tif]

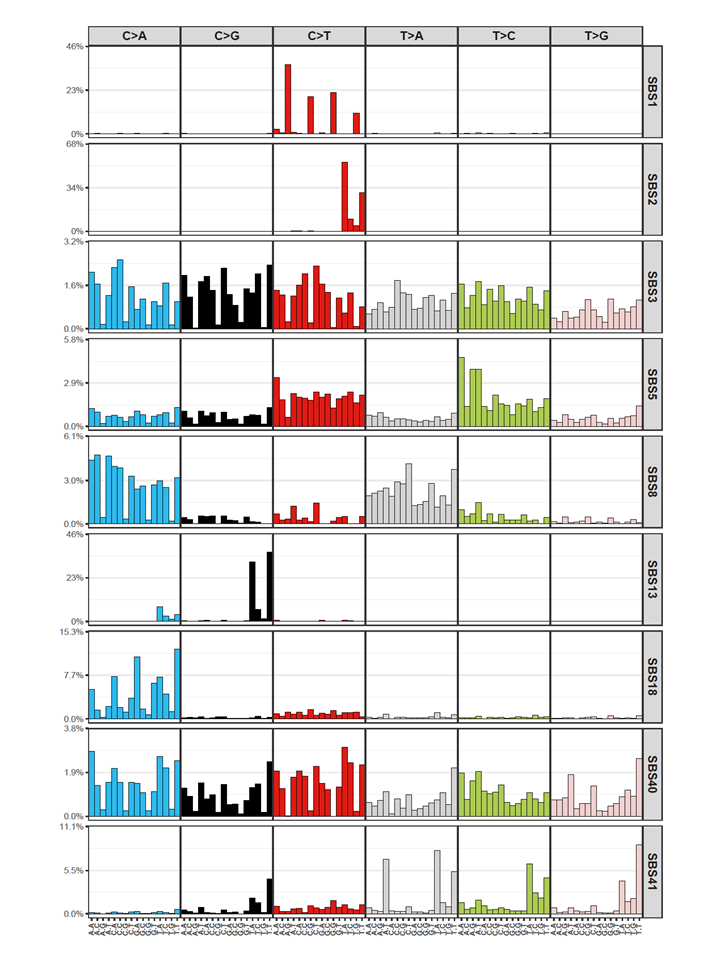

Supplement: S8 Fig — Some signature profiles are spiky (e.g., SBS1 and SBS2/13), while others are relatively flat (e.g., SBS5). (TIF) [file pcbi.1009309.s008.tif]

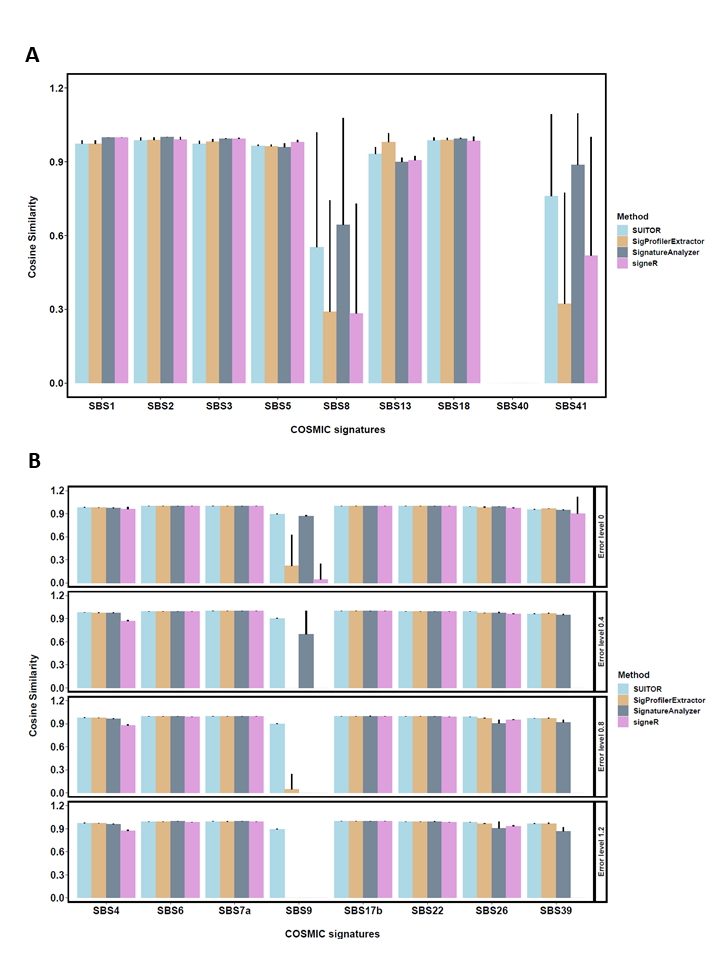

Supplement: S9 Fig — The length of the solid line represents the standard deviation. (TIF) [file pcbi.1009309.s009.tif]

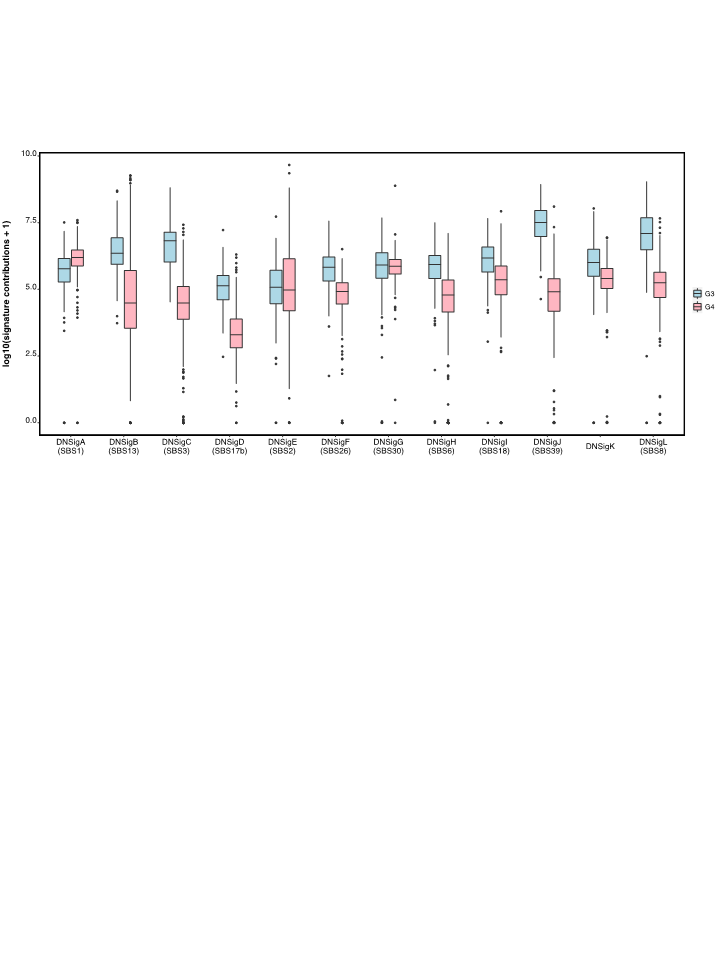

Supplement: S10 Fig — The signatures of the Sanger BRCA study are annotated by COSMIC signatures (if cosine similarities > 0.8) among parentheses. For example, DNSigA (SBS1) refers to de novo signature A (DNSigA) being annotated by COSMIC signature SBS1. (TIF) [file pcbi.1009309.s010.tif]

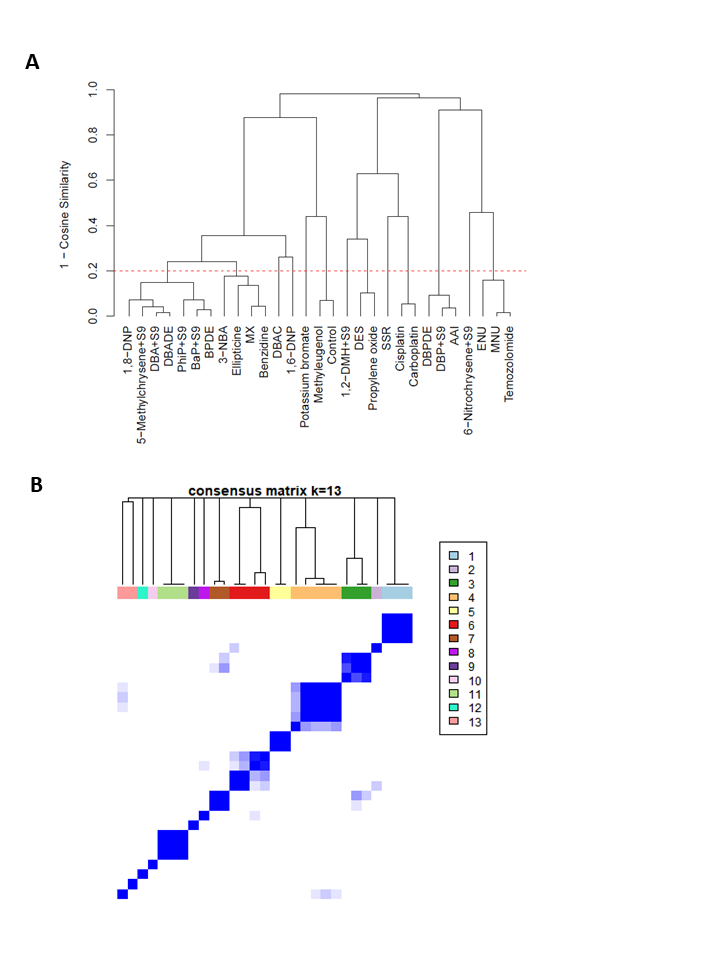

Supplement: S11 Fig — (A) The dendrogram of hierarchical clustering of 28 mutagens with complete linkage method. The height of the branches represents the dissimilarity defined as 1 minus cosine similarity between two signature profiles. The dashed red line corresponds to 0.8 cosine similarity which suggests 13 clusters. (B) The heatmap of the cluster consensus matrix for k = 13. Elements of the consensus matrix show the proportions of concordant mutagen pairs over resampled data: white (0%) indicates never clustered together and dark blue (100%) shows clustered together always. (TIF) [file pcbi.1009309.s011.tif]
