# Supplementary material for: SUITOR: Selecting the number of mutational signatures through cross-validation
Source: PLoS Comput Biol. 2022 Apr 4;18(4):e1009309. doi: 10.1371/journal.pcbi.1009309 (PMC9009674; doi:10.1371/journal.pcbi.1009309)
Supplement: S1 Text — Supplementary note 1 in S1 Text: on Bayesian information criteria (BIC) for mutational signature analysis. Supplementary note 2 in S1 Text: on equivalence between multiplicative update algorithm of NMF and exception/conditional maximization (ECM) algorithm for a Poisson NMF model. Supplementary note 3 in S1 Text: expectation/conditional maximization (ECM) algorithm. (DOCX) [file pcbi.1009309.s012.docx]

**Supplementary note 1: on Bayesian information criteria (BIC) for mutational signature analysis**

A few concerns may arise when applying BIC as a model selection method for mutational signature analysis. First, counting the number of parameters for BIC would be tricking. The number of parameters for BIC is derived from the Laplace approximation to approximate the posterior probability of a model, which tacitly assumes the parameters follow a multivariate normal distribution. However, this distribution would not be applicable to mutational signature analysis, since the parameters corresponding to signature activities and profiles are defined to be non-negative and frequently estimated to be zero. Hence, the multivariate normal distribution would not approximate the distribution of parameters of signature activities and profiles well. Consequently, the specification of number of parameters for BIC is not straightforward for mutational signature analysis. Second, the model selection consistency property [1] of BIC, which is enjoyed by supervised learning (e.g., regression and classification where the number of parameters is fixed), does not hold for mutational signature analysis as a type of unsupervised learning, for which the number of parameters increases with sample size [2]. For these reasons, it’s unclear if BIC is applicable to model selection for mutational signature analysis without major modifications.

**Supplementary note 2: on equivalence between multiplicative update algorithm of NMF and exception/conditional maximization (ECM) algorithm for a Poisson NMF model**

Based on a NMF Poisson model for $v_{pn}$ with $E\left( v_{pn} \right)=\sum_{j=1}^{r} w_{pj}h_{jn}$, $v_{pn}$ can be regarded as the sum of independent latent variables $z_{j,pn}$’s, for which $z_{j,pn}$ follows a Poisson distribution with mean $E\left( z_{j,pn} \right)=w_{pj}h_{jn}$. Hence, it is natural to consider an EM-type algorithm to estimate the latent variables [3].

To derive the EM-type algorithm, we note that the complete data log-likelihood is $\sum_{n,p} \sum_{j=1}^{r} \left\{ -w_{pj}h_{jn}+z_{j,pn}\log\left( w_{pj}h_{jn} \right)-\log\left( z_{j,pn}! \right) \right\}$; the conditional distribution of $z_{j,pn}$ given $v_{pn}$ and the parameters $\mathbf{W}^{\boldsymbol{t}}$ and $\mathbf{H}^{\boldsymbol{t}}$ of the previous step $t$ is the multinomial distribution with the success probability $w_{pj}h_{jn}/\sum_{j=1}^{r} w_{pj}h_{jn}$. Therefore, the E-step evaluates the following quantity

$$Q\left( \mathbf{W},\mathbf{H} | \mathbf{W}^{\boldsymbol{t}},\mathbf{H}^{\boldsymbol{t}} \right)= \sum_{n,p} \sum_{j=1}^{r} \left\{ -w_{pj}h_{jn}+\log\left( w_{pj}h_{jn} \right)v_{pn}\frac{w_{pj}^{t}h_{jn}^{t}}{\sum_{j=1}^{r} w_{pj}^{t}h_{jn}^{t}} \right\}.$$

Next, since it is computationally infeasible to maximize $Q\left( \mathbf{W},\mathbf{H} | \mathbf{W}^{\boldsymbol{t}},\mathbf{H}^{\boldsymbol{t}} \right)$ with respect to $\mathbf{W}$ and $\mathbf{H}$ simultaneously in the M-step, we replace the M-step with two conditional maximization steps. That is, we find a maximizer $\mathbf{W}^{\boldsymbol{t+1}}$ of $Q\left( \mathbf{W},\mathbf{H}^{\boldsymbol{t}} | \mathbf{W}^{\boldsymbol{t}},\mathbf{H}^{\boldsymbol{t}} \right)$ and $\mathbf{H}^{\boldsymbol{t+1}}$ of $Q\left( \mathbf{W}^{\boldsymbol{t+1}},\mathbf{H} | \mathbf{W}^{\boldsymbol{t}},\mathbf{H}^{\boldsymbol{t}} \right)$ sequentially. Specifically, the derivative of

$\frac{\partial}{\partial w_{pj}}Q\left( \mathbf{W},\mathbf{H}^{\boldsymbol{t}} | \mathbf{W}^{\boldsymbol{t}},\mathbf{H}^{\boldsymbol{t}} \right)=-\sum_{n} h_{jn}^{t}+\frac{1}{w_{pj}}\left( \sum_{n} v_{pn}\frac{w_{pj}^{t}h_{jn}^{t}}{\sum_{j=1}^{r} w_{pj}^{t}h_{jn}^{t}} \right)$,

implies

$$w_{pj}^{t+1}=w_{pj}^{t}\frac{\sum_{n} h_{jn}^{t}\left( \frac{v_{pn}}{\sum_{j=1}^{r} w_{pj}^{t}h_{jn}^{t}} \right)}{\sum_{n} h_{jn}^{t}}.$$

Similarly, we obtain

$$h_{jn}^{t+1}=h_{jn}^{t}\frac{\sum_{p} w_{pj}^{t+1}\left( \frac{v_{pn}}{\sum_{j=1}^{r} w_{pj}^{t+1}h_{jn}^{t}} \right)}{\sum_{p} w_{pj}^{t+1}}.$$

Notably, these EM steps coincide with the multiplicate update algorithm [4].

**Supplementary note 3: expectation/conditional maximization (ECM) algorithm**

We outline below the full steps for SUITOR. We denote [$minR, maxR$] as the range of number of signatures to be examined, $\boldsymbol{\circ}$ the dot product, p the pth mutation type and n the nth tumor. We try $I$ random initial $\mathbf{W}^{\boldsymbol{0}}$ and $\mathbf{H}^{\boldsymbol{0}}$ to mitigate the possibility that the ECM algorithm will converge to local saddle points.

| **ECM algorithm full steps for SUITOR** |
| --- |

Divide index matrix $\mathcal{S}$ into $K$ disjoint sets $\mathcal{S}_{1}, \cdots,\mathcal{S}_{K}$

**for** $r$ = $minR$ to $maxR$ do

**for** $k$ = 1 to $K$ do

Reserve validation set: $\mathbf{V}_{k}^{L}=\left\{ v_{pn}|(n,p)\in\mathcal{S}_{k} \right\}$

Choose initial values $\mathbf{M}_{k}^{0}$ as medians of available mutation counts in the same row of $\mathbf{V}_{k}^{T}$for a mutation type.

**for** $i$ = 1 to $I$

set initial $\mathbf{W}^{\boldsymbol{0}}$ and $\mathbf{H}^{\boldsymbol{0}}$ from a NMF algorithm for $(\mathbf{V}_{k}^{T}, \mathbf{M}_{k}^{0})$ with rank $r$ and seed $i$

**iterate** with respect to $t$:

E-step: given $\mathbf{W}^{\boldsymbol{t}}$ and $\mathbf{H}^{\boldsymbol{t}}$, update elements of $\mathbf{M}_{k}^{t}$ with $\mathbf{W}^{\boldsymbol{t}}\mathbf{H}^{\boldsymbol{t}}$ for $\left( n,p \right)\in\mathcal{S}_{k}$

CM1-step: update parameters $\mathbf{W}^{\boldsymbol{t+1}}$ via

$$\mathbf{W}^{\boldsymbol{t+1}}\boldsymbol{\leftarrow}\mathbf{W}^{\boldsymbol{t}}\boldsymbol{\circ}\frac{{\frac{\boldsymbol{V}_{\boldsymbol{k}}}{\mathbf{W}^{\boldsymbol{t}}\mathbf{H}^{\boldsymbol{t}}}\boldsymbol{(}\mathbf{H}^{\boldsymbol{t}}\boldsymbol{)}}^{T}}{{\boldsymbol{1}_{\boldsymbol{N\times96}}\boldsymbol{(}\mathbf{H}^{\boldsymbol{t}}\boldsymbol{)}}^{T}}$$

CM2-step: update parameters $\mathbf{H}^{\boldsymbol{t+1}}$ via

$$\mathbf{H}^{\boldsymbol{t+1}}\boldsymbol{\leftarrow}\mathbf{H}^{\boldsymbol{t}}\boldsymbol{\circ}\frac{{\boldsymbol{(}\mathbf{W}^{\boldsymbol{t+1}}\boldsymbol{)}}^{T}\frac{\boldsymbol{V}_{\boldsymbol{k}}}{\mathbf{W}^{\boldsymbol{t+1}}\mathbf{H}^{\boldsymbol{t}}}}{{\boldsymbol{(}\mathbf{W}^{\boldsymbol{t+1}}\boldsymbol{)}}^{T} \boldsymbol{1}_{\boldsymbol{N\times96}}}$$

**until** $\left| \frac{\log\left\{ \Pr\left( \mathbf{V}_{k}^{T},\mathbf{M}_{k}^{t+1} | \mathbf{W}^{t+1}\mathbf{H}^{t+1} \right) \right\}-\log\left\{ \Pr\left( \mathbf{V}_{k}^{T},\mathbf{M}_{k}^{t} | \mathbf{W}^{t}\mathbf{H}^{t} \right) \right\}}{\log\left\{ \Pr\left( \mathbf{V}_{k}^{T},\mathbf{M}_{k}^{t} | \mathbf{W}^{t}\mathbf{H}^{t} \right) \right\}} \right|\mathbf{<}\epsilon$ or iteration exceeds max.iter

Record the training error ${TR}_{r,k,i}=-log \left\{ \Pr\left( \mathbf{V}_{k}^{T} | \hat{\mathbf{W}}\hat{\mathbf{H}} \right) \right\}$ and

the validation error ${ERR}_{r,k,i}=-log \left\{ \Pr\left( \mathbf{V}_{k}^{L} | {\hat{\boldsymbol{M}}}_{k} \right) \right\}$

**end for**

Find $i_{r,k}^{*}$= $\underset{1\leq i\leq I}{\mathrm{argmax}} {TR}_{r,k,i}$ and save ${ERR}_{r,k,i^{*}}$

**end for**

Define cross validation error ${ERR}_{r,k}=\sum_{k} {ERR}_{r,k,i^{*}}$

**end for**

find $r^{*}=\underset{1\leq r\leq R}{\mathrm{argmax}} {ERR}_{r,k}$

|  |
| --- |

**Reference:**

1. Nishii, R. Asymptotic properties of criteria for selection of variables in multiple regression. *The Annals of Statistics*, 758-765 (1984).

2. Shao, J. An asymptotic theory for linear model selection. *Statistica sinica*, 221-242 (1997).

3. Févotte, C. & Cemgil, A.T. Nonnegative matrix factorizations as probabilistic inference in composite models. in *2009 17th European Signal Processing Conference* 1913-1917 (2009).

4. Lee, D.D. & Seung, H.S. Learning the parts of objects by non-negative matrix factorization. *Nature* **401**, 788-91 (1999).
