# Supplementary material for: SUITOR: Selecting the number of mutational signatures through cross-validation
Source: PLoS Comput Biol. 2022 Apr 4;18(4):e1009309. doi: 10.1371/journal.pcbi.1009309 (PMC9009674; doi:10.1371/journal.pcbi.1009309)
Supplement: S1 Data — (DOCX) [file pcbi.1009309.s014.docx]

**Software and dataset webpages:**

COSMIC mutational signatures (V3.2-March 2021): <https://cancer.sanger.ac.uk/signatures/>

SUITOR: <https://github.com/binzhulab/SUITOR>

SigProfilerExtractor: v1.0.6 is installed and used by following instructions in <https://github.com/AlexandrovLab/SigProfilerExtractor>.

SignatureAnalyzer (downloaded March 2020): version information is not available but is installed by following instructions in <https://github.com/broadinstitute/getzlab-SignatureAnalyzer>. SignatureAnalyzer was run based on CPU (not GPU).

signeR: v1.12.0 is downloaded from <https://bioconductor.org/packages/release/bioc/html/signeR.html>.

CV2K (downloaded September 2021): <https://github.com/GalGilad/CV2K>

SparseSignatures (version 2.4.0): <https://www.bioconductor.org/packages/release/bioc/html/SparseSignatures.html>

SigProfilerExtractor and SignatureAnalyzer are used in python version 3.7.5 while SUITOR and signeR are used in R version 3.6.3. Signature profiles plot is drawn based on R package MutationalPatterns from <https://bioconductor.org/packages/release/bioc/html/MutationalPatterns.html> with slight modification to add graphical parameters.

Data for In vitro studies
http://medgen.medschl.cam.ac.uk/serena-nik-zainal/

Data for Sanger whole genome sequencing breast cancer study
ftp://ftp.sanger.ac.uk/pub/cancer/Nik-ZainalEtAl-560BreastGenomes
(Note: it’s an FTP site. For Mac OS, please use the Finder window to access it; for Windows, use the File Explorer in Windows 8, or Windows Explorer in previous versions; the FTP site can also be accessed by FTP Clients, such as FileZilla, WinSCP and CyberDuck.)

Data for The Pan-Cancer Analysis of Whole Genomes (PCAWG) study
[https://www.synapse.org/#!Synapse:syn11726620](https://www.synapse.org/#!Synapse:syn11726620)
