# Supplementary material for: SUITOR: Selecting the number of mutational signatures through cross-validation
Source: PLoS Comput Biol. 2022 Apr 4;18(4):e1009309. doi: 10.1371/journal.pcbi.1009309 (PMC9009674; doi:10.1371/journal.pcbi.1009309)

Supplementary Figures

Supplementary Figure 1: The number of signatures selected for the knockout study of DNA repair gene *MSH6.* Each plot shows how sigProfiler (a), signatureAnalyzer (b) and signeR (c) select the optimal number of signatures respectively.

Supplementary Figure 2: The signatures detected in knockout studies of six DNA repair genes. a) The plots of criteria to select the optimal number of signatures by SUITOR, sigProfiler, signeR and signatureAnalyzer (in clockwise order). b) The profiles of signatures discovered by each method.

Supplementary Figure 3: Signature profiles identified experimentally from the *in vitro* study of environmental or therapeutic mutagens. The signatures include 28 mutagen-induced signatures and a background signature existing in control samples and all mutagen treated samples. ENU: N-ethyl-N-nitrosourea; DBP+S9: dibenzo[a,l]pyrene mixed with S9 rodent liver-derived metabolic enzyme; PhiP+S9: 2-amino-1-methyl-6-phenylimidazo[4,5-b]pyridine mixed with S9 rodent liver-derived metabolic enzyme; MNU: N-methyl-N-nitrosourea; DBPDE: dibenzo[a,l]pyrene diol-epoxide; MX: 3-chloro-4-(dichloromethyl)-5-hydroxy- 2(5H)-furanone; AAI: aristolochic acid I; 1,2-DMH+S9: 1,2-dimethylhydrazine mixed with S9 rodent liver-derived metabolic enzyme; 1,8-DNP: 1,8-Dinitropyrene; DBA+S9: dibenz[a,h]anthracene mixed with S9 rodent liver-derived metabolic enzyme; 3-NBA: 3-nitrobenzanthrone; DBADE: dibenz[a,h]anthracene diol-epoxide; 1,6-DNP: 1,6-Dinitropyrene; DES: diethyl sulfate; DBAC: dibenz[a,j]acridine; 5-Methylchrysene+S9: 5-Methylchrysene mixed with S9 rodent liver-derived metabolic enzyme; SSR: simulated solar radiation; BaP+S9: benzo[a]pyrene mixed with S9 rodent liver-derived metabolic enzyme; 6-Nitrochrysene+S9: 6-Nitrochrysene mixed with S9 rodent liver-derived metabolic enzyme; BPDE: benzo[a]pyrene-7,8-dihydrodiol-9,10-epoxide.

Supplementary Figure 4: The number of signatures selected from the *in vitro* study of environmental or therapeutic mutagens by a) sigProfiler, b) signatureAnalyzer and c) signeR . The numbers of detected signatures include one background signature and additional mutagen-induced mutational signatures.

Supplementary Figure 5: Clusters of subclones of *in vitro* study of exogenous mutagens, visualized by t-SNE for SUITOR, sigProfiler, signatureAnalyzer and signeR. Each dot represents a subclone, colored by the mutagen treatment.

Supplementary Figure 6: The number of signatures selected for in silico simulation studies with one signature by SUITOR (a), sigProfiler (b), signatureAnalyzer (c) and signeR (d) for one replicate as an illustration.

Supplementary Figure 7: The profiles of nine signatures pre-specified in the *in silico* simulation studies. Some signature profiles are spiky (e.g., SBS1 and SBS2/13), while others are relatively flat (e.g., SBS5).

Supplementary Figure 8: The boxplots of mutation contributions of 12 signatures for signature clusters G3 and G4 in Sanger breast cancer study.

Supplementary Figure 1

a

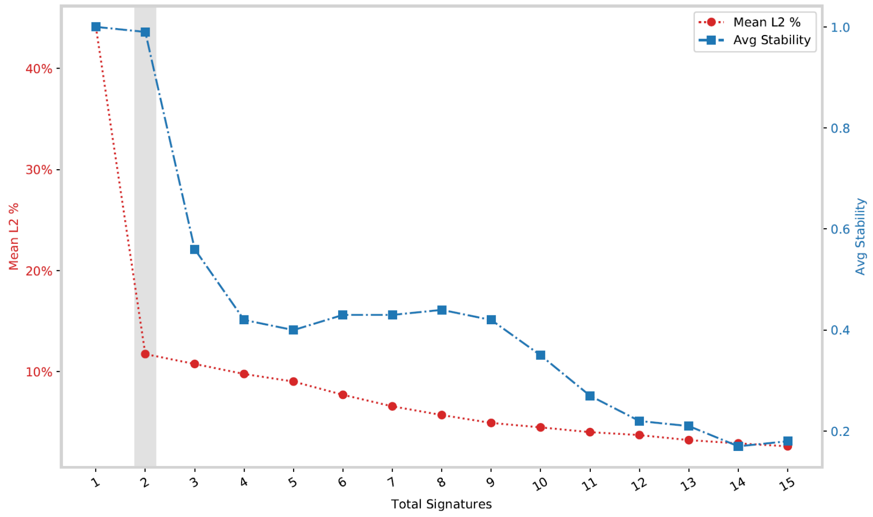

b

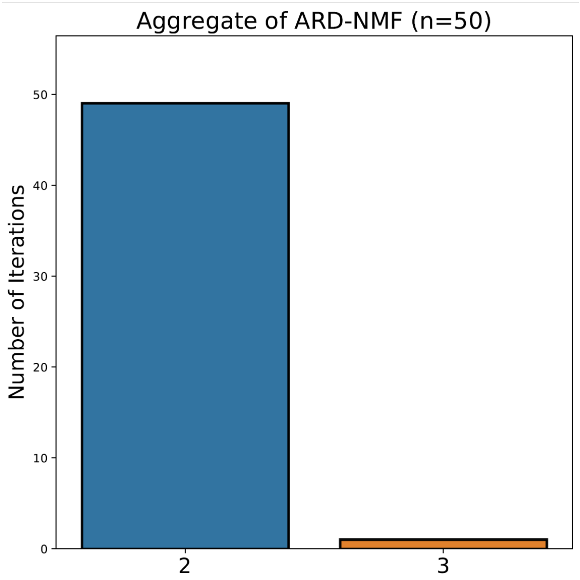

c

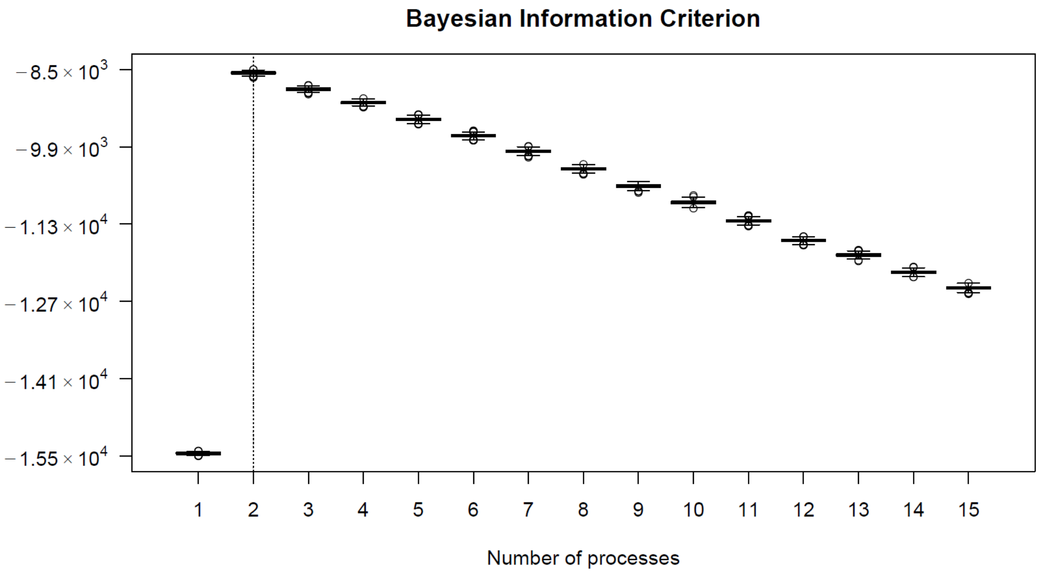

Supplementary Figure 2

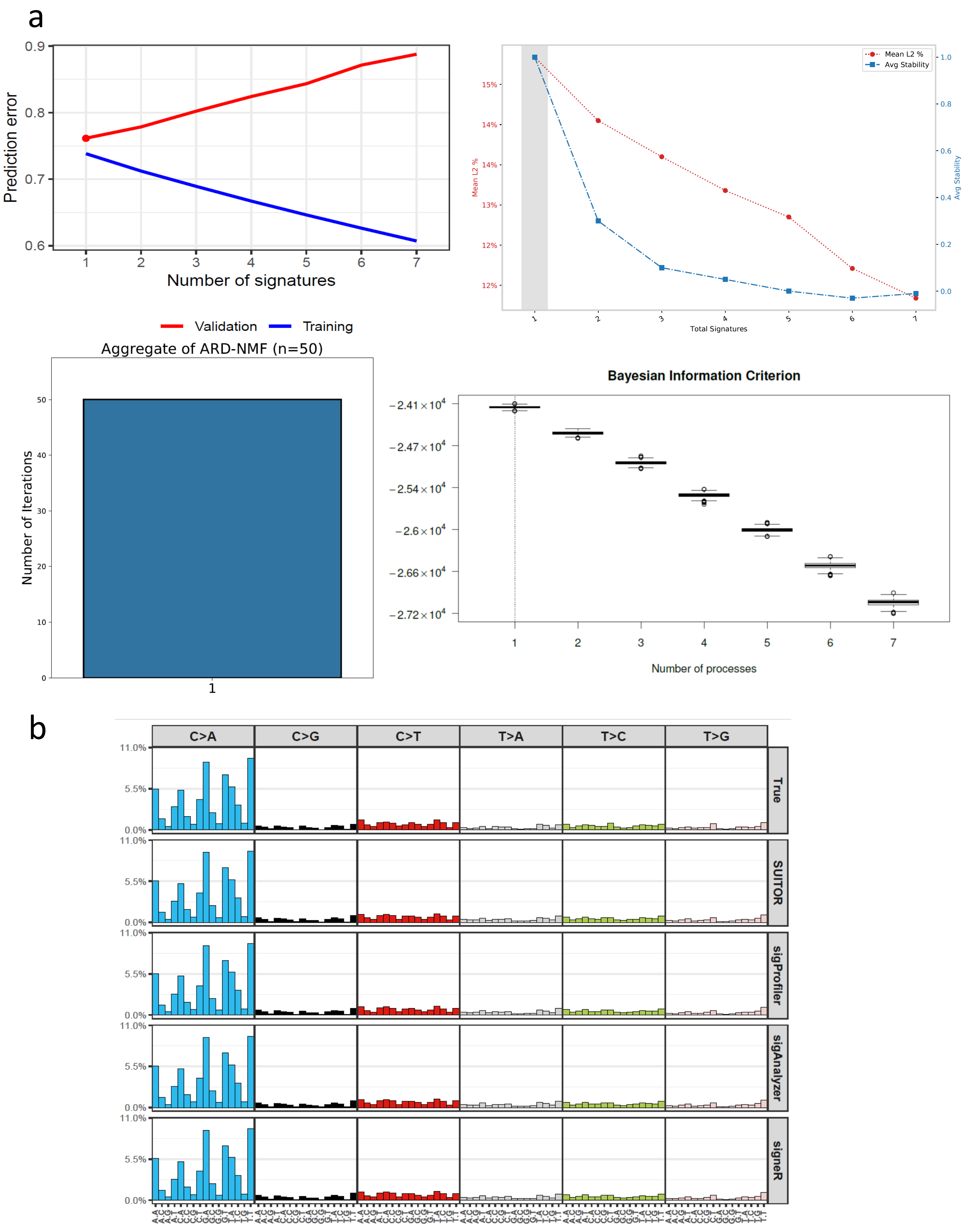

Supplementary Figure 3

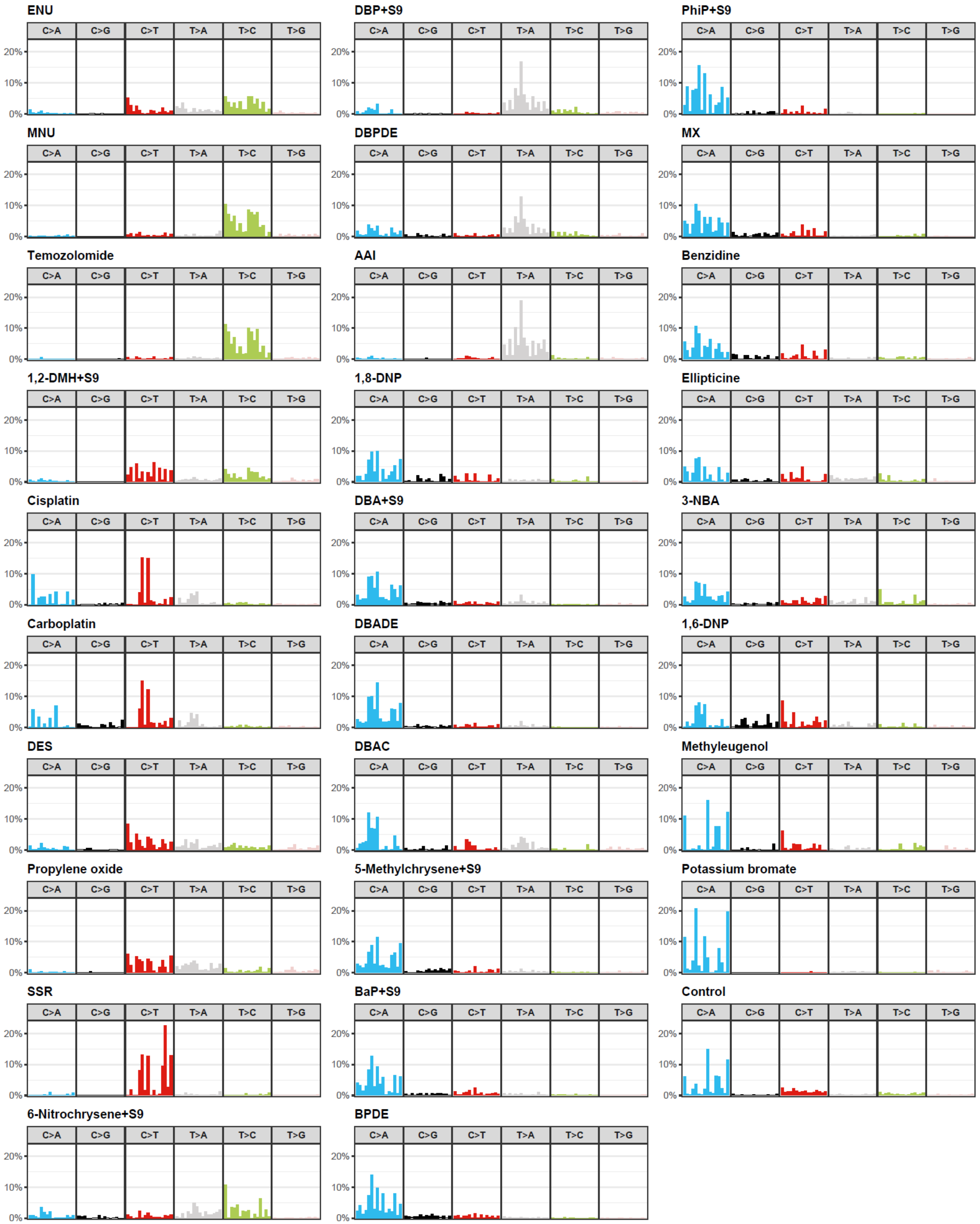

Supplementary Figure 4

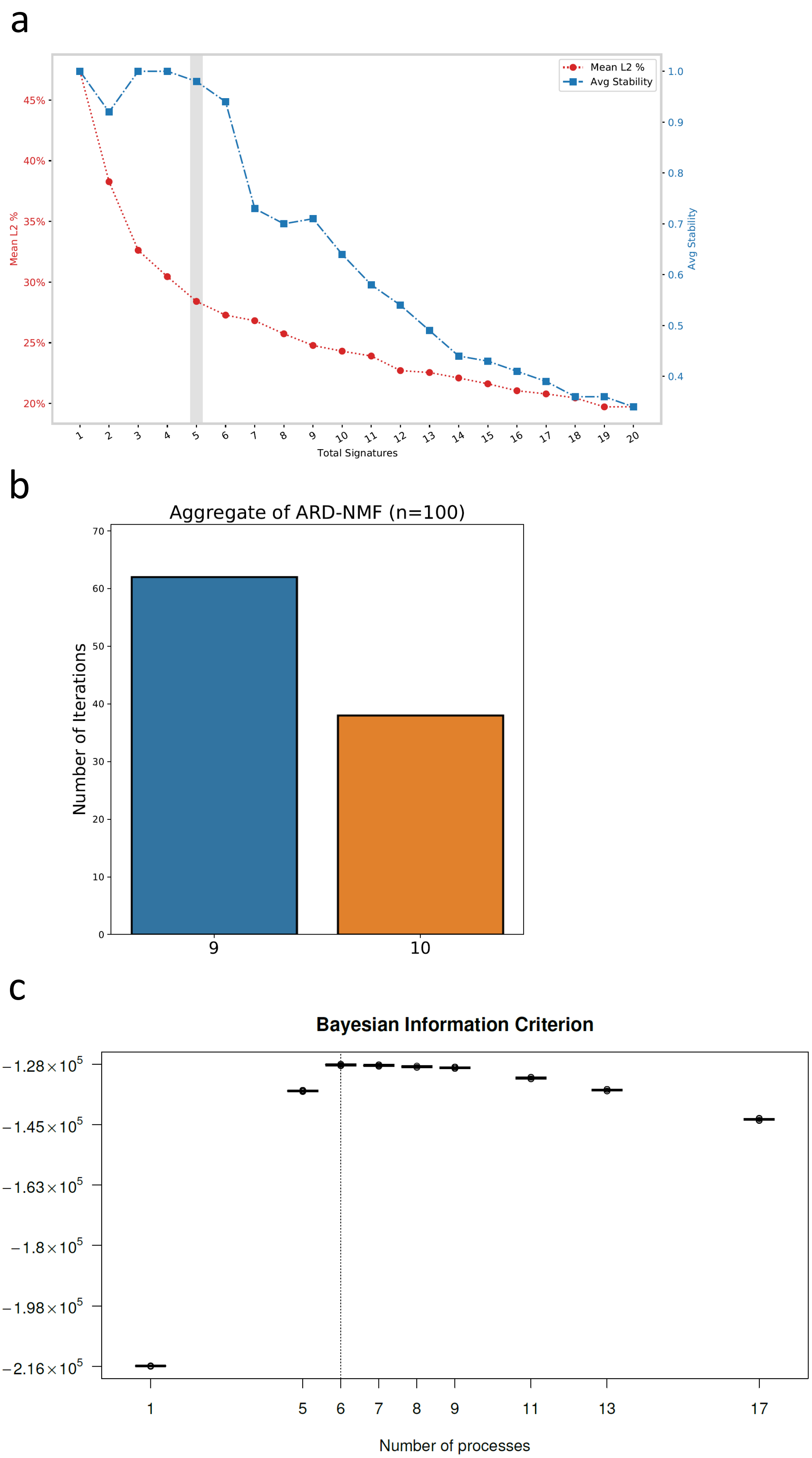

Supplementary Figure 5

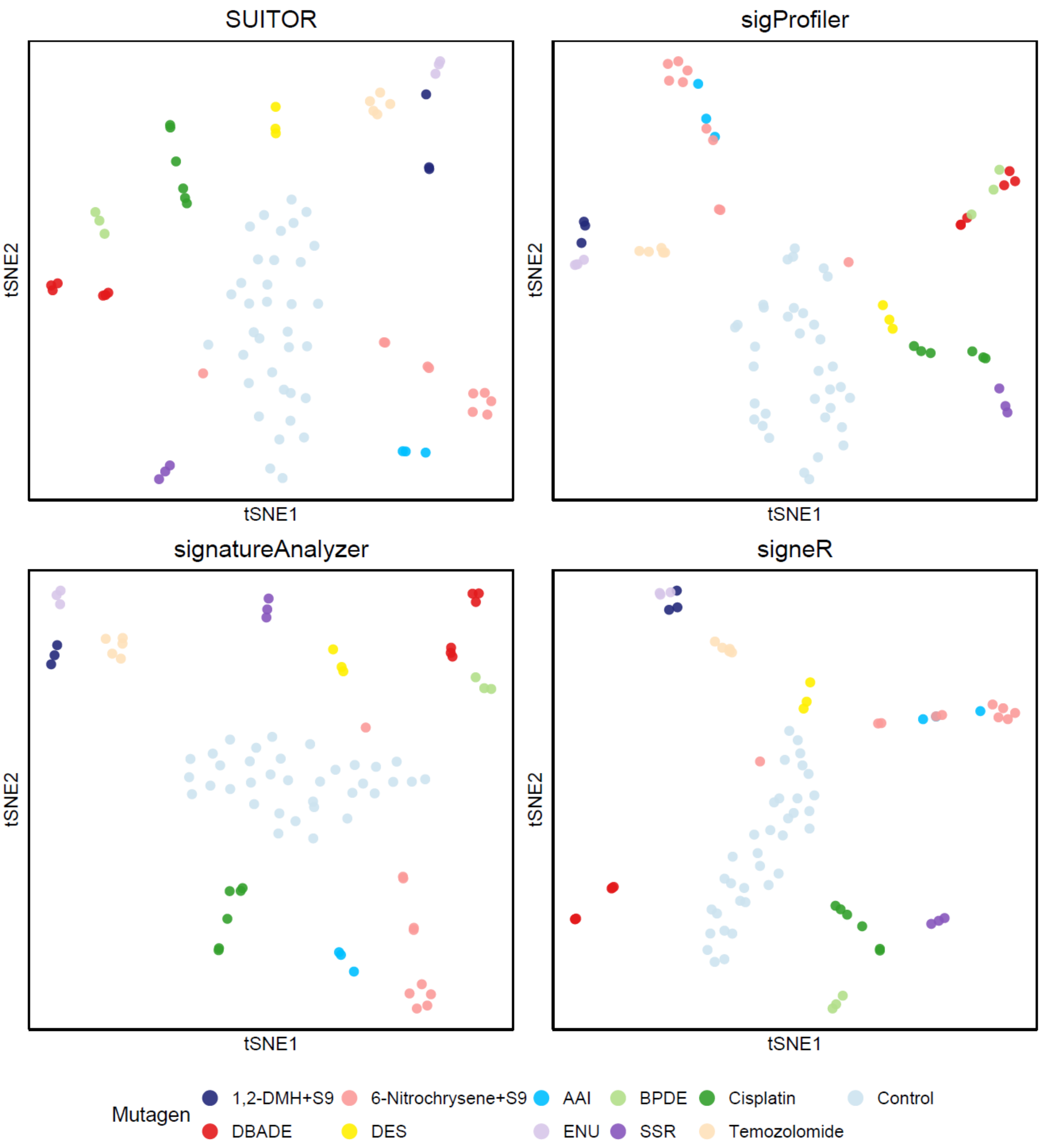

Supplementary Figure 6

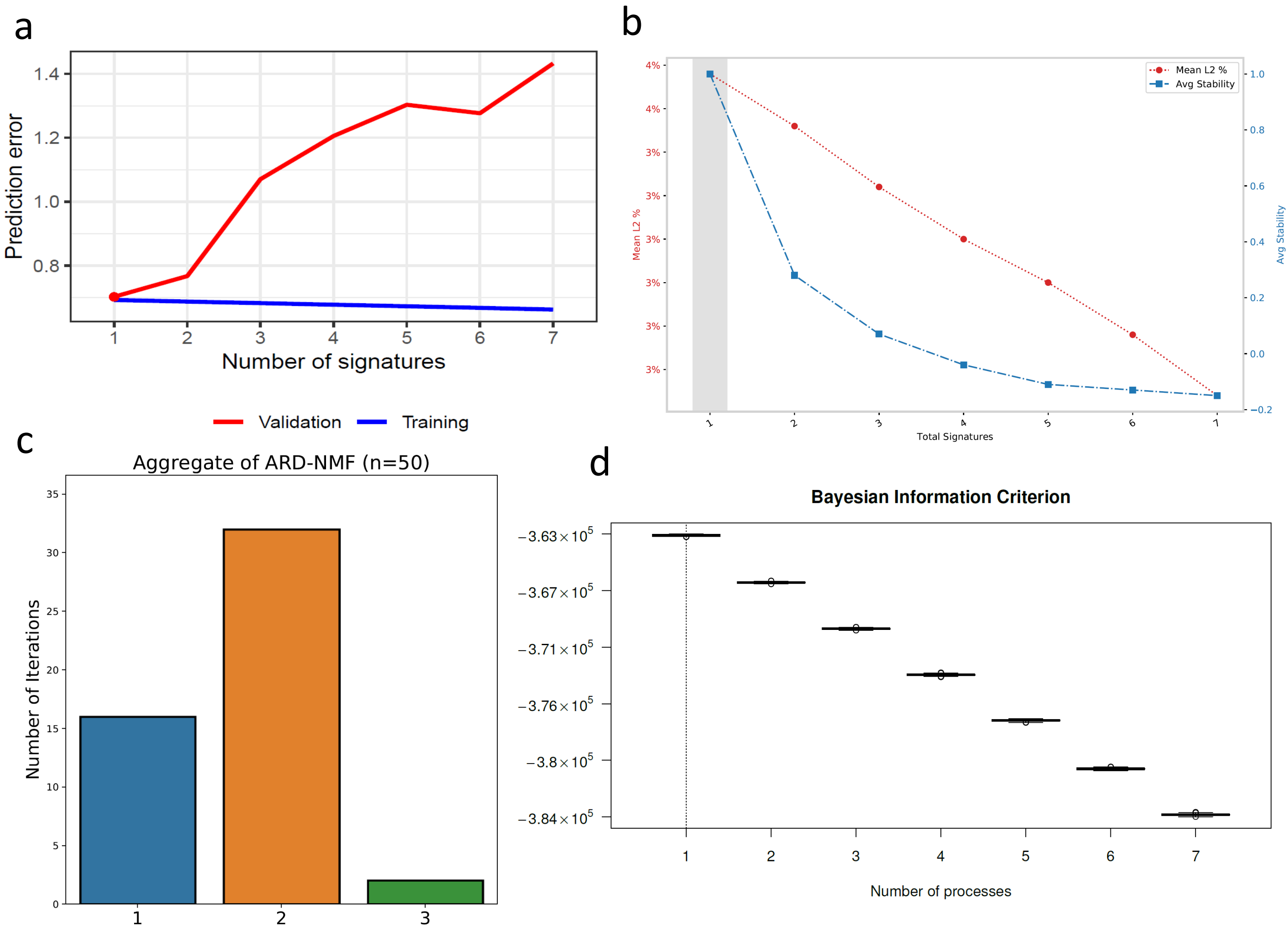

Supplementary Figure 7

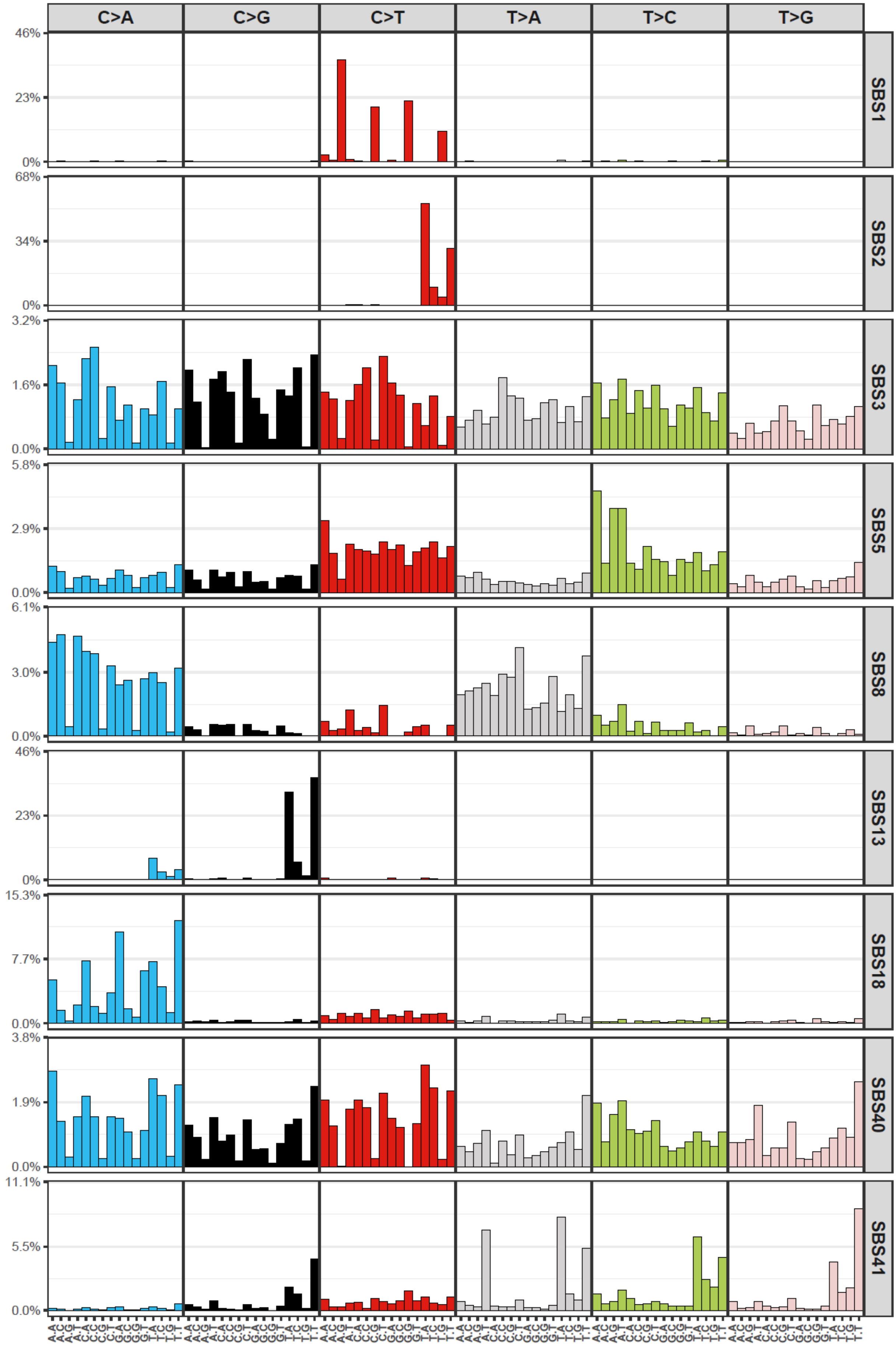

Supplementary Figure 8

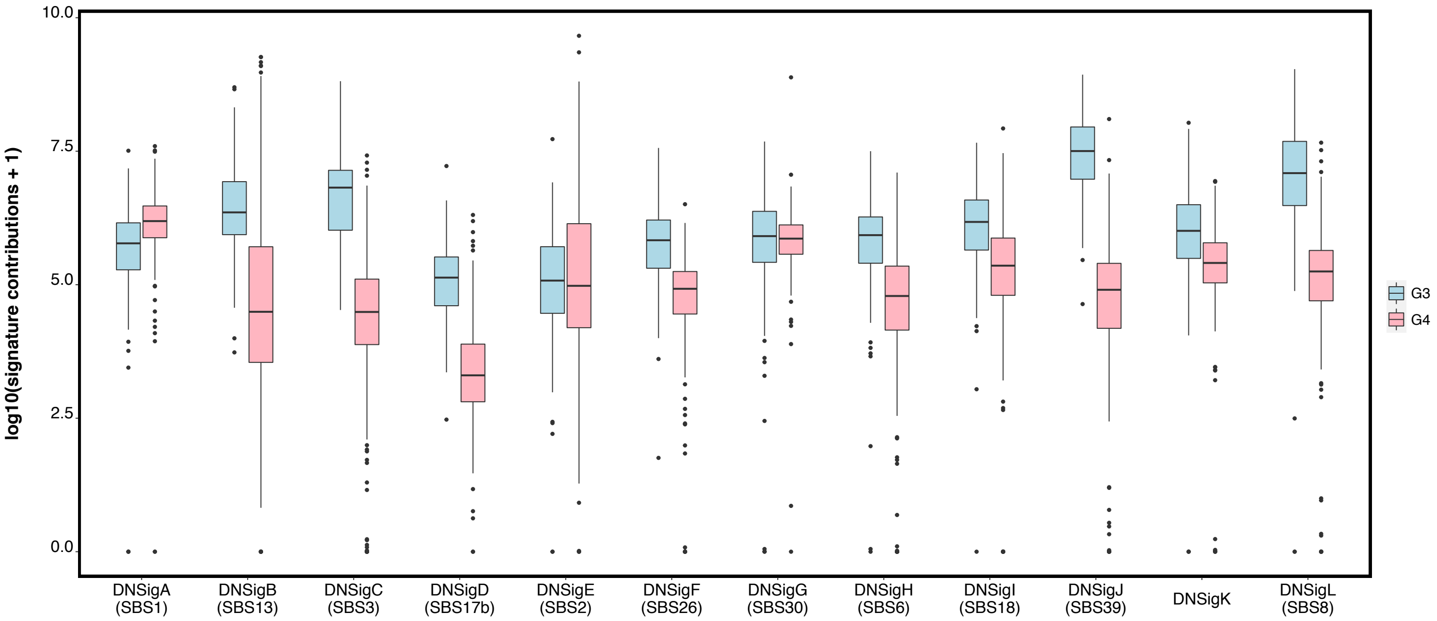

Supplement: Attachment — Submitted filename: Supplementary figures.pdf [file pcbi.1009309.s016.pdf]
