# Supplementary material for: SUITOR: Selecting the number of mutational signatures through cross-validation
Source: PLoS Comput Biol. 2022 Apr 4;18(4):e1009309. doi: 10.1371/journal.pcbi.1009309 (PMC9009674; doi:10.1371/journal.pcbi.1009309)
Supplement: Attachment — Submitted filename: PCOMPBIOL-D-21-01288.pdf [file pcbi.1009309.s017.pdf]

PLOS Computational Biology

SUITOR: selecting the number of mutational signatures through cross-validation

--Manuscript Draft--

**Manuscript Number:**	PCOMPBIOL-D-21-01288
**Full Title:**	SUITOR: selecting the number of mutational signatures through cross-validation
**Short Title:**	Selecting the number of mutational signatures
**Article Type:**	Research Article
**Keywords:**	Cancer genomics; cross-validation; mutational signatures; whole genome sequencing
**Abstract:**	For de novo mutational signature analysis, the critical first step is to decide how many signatures should be expected in a cancer genomics study. An incorrect number could mislead downstream analyses. Here we present SUITOR (Selecting the nUmber of mutatIonal signaTures thrOugh cRoss-validation), an unsupervised cross-validation method that requires little assumptions and no numerical approximations to select the optimal number of signatures without overfitting the data. In vitro studies and in silico simulations demonstrated that SUITOR can correctly identify signatures, some of which were missed by other widely used methods. Applied to 2,540 whole-genome sequenced tumors across 22 cancer types, SUITOR selected signatures with the smallest prediction errors and almost all signatures of breast cancer selected by SUITOR were validated in an independent breast cancer study. SUITOR is a powerful tool to select the optimal number of mutational signatures, facilitating downstream analyses with etiological or therapeutic importance.
**Additional Information:**	
**Question**	**Response**
**Financial Disclosure**<br><br>Enter a financial disclosure statement that describes the sources of funding for the work included in this submission. Review the submission guidelines for detailed requirements. View published research articles from *PLOS Computational Biology* for specific examples.<br><br>This statement is required for submission and **will appear in the published article** if the submission is accepted. Please make sure it is accurate.	The author(s) received no specific funding for this work.

Powered by Editorial Manager® and ProduXion Manager® from Aries Systems Corporation

**Unfunded studies**<br>Enter: *The author(s) received no specific funding for this work.*<br><br>**Funded studies**<br>Enter a statement with the following details:<br>• Initials of the authors who received each award<br>• Grant numbers awarded to each author<br>• The full name of each funder<br>• URL of each funder website<br>• Did the sponsors or funders play any role in the study design, data collection and analysis, decision to publish, or preparation of the manuscript?<br>• **NO** - Include this sentence at the end of your statement: *The funders had no role in study design, data collection and analysis, decision to publish, or preparation of the manuscript.*<br>• **YES** - Specify the role(s) played.<br><br>* typeset	
**Competing Interests**<br><br>Use the instructions below to enter a competing interest statement for this submission. On behalf of all authors, disclose any competing interests that could be perceived to bias this work—acknowledging all financial support and any other relevant financial or non-financial competing interests.<br><br>This statement **will appear in the published article** if the submission is accepted. Please make sure it is accurate. View published research articles from *PLOS Computational Biology* for specific examples.	The authors have declared that no competing interests exist.

**NO authors have competing interests**<br><br>Enter: *The authors have declared that no competing interests exist.*<br><br>**Authors with competing interests**<br><br>Enter competing interest details beginning with this statement:<br><br>*I have read the journal's policy and the authors of this manuscript have the following competing interests: [insert competing interests here]*<br><br>* typeset<br><br>This statement is required for submission and **will appear in the published article** if the submission is accepted. Please make sure it is accurate and that any funding sources listed in your Funding Information later in the submission form are also declared in your Financial Disclosure statement.	
**Data and Code Availability**<br><br>From the time of publication, Authors are required to make fully available and without restriction all data and computational code underlying their findings. Please see our PLOS Data Policy page for detailed policy information, and our Code Sharing page for specific information on code sharing.<br><br>A **Data Availability Statement**, detailing where the data (and code, if applicable) can be accessed, is required at first submission. Insert your Data Availability Statement in the box below. The statement you provide **will be published in the article**, if accepted.<br><br>Please see the Data Reporting section of our submission guidelines for instructions on what you need to include in your Data Availability Statement.	The data used are public available. The download links are specified in the Method section.

Powered by Editorial Manager® and ProduXion Manager® from Aries Systems Corporation

If the data and code are all contained in your submission files, please state: *All relevant data are within the manuscript and its Supporting Information files.*<br><br>**PLOS allows rare exemptions to address legal and ethical concerns.** If you have legal or ethical restrictions, please detail these in your Data Availability Statement below for the Journal team to consider.	

Powered by Editorial Manager® and ProduXion Manager® from Aries Systems Corporation

Manuscript
Click here to access/download;Manuscript;Main_with_methods.docx

SUITOR: selecting the number of mutational signatures through cross-validation

Donghyuk Lee[1], Difei Wang[2], Xiaohong R. Yang[2], Jianxin Shi[2], Maria Teresa Landi[2], Bin Zhu[2,#]

[1]Department of Statistics, Pusan National University, Busan, Korea

[2]Division of Cancer Epidemiology and Genetics, National Cancer Institute, National Institutes of Health, Bethesda, MD, USA

# To whom correspondence should be addressed to: bin.zhu@nih.gov

Abstract

For *de novo* mutational signature analysis, the critical first step is to decide how many signatures should be expected in a cancer genomics study. An incorrect number could mislead downstream analyses. Here we present SUITOR (Selecting the nUmber of mutatIonal signaTures thrOugh cRoss-validation), an unsupervised cross-validation method that requires little assumptions and no numerical approximations to select the optimal number of signatures without overfitting the data. *In vitro* studies and *in silico* simulations demonstrated that SUITOR can correctly identify signatures, some of which were missed by other widely used methods. Applied to 2,540 whole-genome sequenced tumors across 22 cancer types, SUITOR selected signatures with the smallest prediction errors and almost all signatures of breast cancer selected by SUITOR were validated in an independent breast cancer study. SUITOR is a powerful tool to select the optimal number of mutational signatures, facilitating downstream analyses with etiological or therapeutic importance.

Background

Mutational signatures are patterns of somatic mutations imprinted on the cancer genome by operative mutational processes. At least 49 single base substitution mutational signatures have been identified across cancer types[1], with some associated with exogenous mutagenic exposures[2-4] and endogenous mutational processes[5-8]. Moreover, mutational signatures have been proposed to identify cancer predisposition genes (e.g., *NTHL1* in breast cancer[9]), and to stratify cancer patients[10-13] for precision treatment. In these studies, deciding the expected number of signatures is the pivotal first step, which determines the downstream steps of extracting signature profiles, estimating signature contributions and stratifying tumors based on signatures for treatment. As an example, the Pan-Cancer Analysis of Whole Genomes (PCAWG) consortium reported that the discordance between the extracted and known signatures is usually caused by the difficulty in selecting the correct number of signatures[1].

Despite the many algorithms proposed to extract signature profiles[14-18] and estimate signature contributions[19,20], relatively little emphasis has been placed on selecting the correct number of *de novo* mutational signatures in cancer genomics studies[21,22]. SomaticSignatures[14] chooses the number of signatures based on the residual sum of squares and the explained variance without a clearly defined selection criterion. SigProfiler[15] considers the mean reconstruction error and the stability of signature extraction; however, it is unclear how these features could be combined to jointly predict the number of signatures. EMu[16] and signeR[17] adopt a Bayesian information criterion (BIC)[23]. Although BIC is a popular model selection criterion for supervised learning (e.g., regression and classification) where the number of parameters is fixed, it may not

be applicable to unsupervised learning, including mutational signature analysis, where the number of parameters increases with the sample size (other limitations of BIC elaborated in Supplementary Note 1). SignatureAnalyzer[1] uses an automatic relevance determination (ARD) prior[24] which imposes a sparsity assumption on mutation profiles and contributions. The number of signatures chosen by signatureAnalyzer is sensitive to the pre-specified sparsity assumption, especially hyperparameters of the ARD prior and the tolerance level.

To overcome the limitations of previous methods, we propose selecting the number of signatures through cross-validation. Selecting the number of signatures is essentially a problem of model selection, which has been addressed by cross-validation in other research areas[25,26], including identification of cancer subtypes[27], exploration of population structure[28] and prediction of lymph node metastasis[29]. In the setting of mutational signature analysis, cross-validation splits the full dataset (here, the mutation counts) into a training set and a validation set; for a given number of signatures, these signatures are estimated in the training set and then they are used to predict the mutations in the validation set. Multiple candidate numbers of signatures are considered; and the number of signatures which predicts most closely the mutations in the validation (not the training) set is selected. Hence, cross-validation can prevent selecting too few or too many signatures (corresponding to an underfitting or overfitting model), both of which would predict mutation counts in the validation set poorly. In addition, unlike the BIC or the ARD prior, cross-validation requires little assumptions and no numerical approximations[26]. Therefore, cross-validation provides a viable solution for selecting the correct number of signatures.

Despite being conceptually appealing, the standard cross-validation approach does not work for unsupervised mutational signature analysis. In the standard cross-validation scheme, it is feasible to remove a subset of subjects all together as a validation set because parameters (e.g., regression coefficients) are identical across subjects. However, mutation contributions in mutational signature analysis are tumor-specific parameters. If mutation counts of a subset of tumors were removed all together as a validation set, these counts would be used twice (for estimating tumor-specific mutation contributions and calculating the prediction error), which would underestimate the prediction error. Instead, cross-validation for mutational signature analysis requires retaining all tumors in the training set but removing some mutation counts from each tumor as a validation set. Consequently, missing data emerge in the training set, which would cause current methods for mutational signature analysis to fail.

These limitations are overcome by SUITOR (Selecting the nUmber of mutatIonal signaTures thrOugh cRoss-validation), an unsupervised cross-validation method that selects the optimal number of signatures to attain the minimal prediction error in the validation set. SUITOR extends the probabilistic model to allow missing data in the training set, which makes cross-validation feasible. Moreover, we propose an expectation/conditional maximization (ECM) algorithm[30] to extract signature profiles, estimate mutation contributions and impute the missing data simultaneously. We demonstrated SUITOR's superior performance using *in vitro* experimental data, *in silico* simulations, *in vivo* applications to 2,540 tumors across 22 cancer types, and validation of signatures of breast cancer in additional 440 breast tumors.

Results

Overview of SUITOR

Mutational signature analysis decomposes the catalog matrix of somatic mutations. Take single base substitution (SBS) as an example. A mutation catalog matrix $\mathbf{V}$ of size $\mathrm{N} \times 96$ contains mutation counts for N cancer genomes and 96 SBS catalogs. Each SBS catalog refers to a mutated pyrimidine (C or T) in the center and two unmutated adjacent nucleotides (flanking 5' and 3' bases) with total $4 \times 6 \times 4 = 96$ catalogs. For example, a genomic sequence A$\underline{\text{C}}$G in the normal tissue is mutated to A$\underline{\text{G}}$G in the tumor tissue. This SBS belongs to the A[C > G]G mutation catalog.

SUITOR is built upon a probabilistic model[31,32], for which the maximum likelihood estimation (MLE) is equivalent to the solution of non-negative matrix factorization (NMF), the most popular method for mutational signature analysis[15]. Given the number of signatures r to be extracted, NMF factorizes the mutation catalog matrix into two non-negative matrices: signature contribution matrix $\mathbf{W}$ of size $\mathrm{N} \times r$ and signature profile matrix $\mathbf{H}$ of size $r \times 96$ such that $\mathbf{V} \approx \mathbf{WH}$. Each row of $\mathbf{W}$ contains attributions of r signatures, reflecting how intense r mutational signatures are in a tumor; each row of $\mathbf{H}$ forms a signature profile with the elements summed to 1, showing how 96 mutation catalogs comprise a signature profile. To estimate $\mathbf{W}$ and $\mathbf{H}$, it is common to minimize the generalized Kullback-Leibler (KL) divergence

$$D_{KL}(\mathbf{V}|\mathbf{WH}) = \sum_{p=1}^{96} \sum_{n=1}^{N} \left\{ v_{np} \log \left({v_{np}} \Big/ {\sum_{j=1}^{r} w_{nj} h_{jp}} \right) + \sum_{j=1}^{r} w_{nj} h_{jp} - v_{np} \right\},$$

subject to $w_{nj} \geq 0$ and $h_{jp} \geq 0$ with $1 \leq j \leq \mathrm{r}$, $1 \leq n \leq \mathrm{N}$ and $1 \leq p \leq 96$. Lowercase letters, v_{np}, w_{nj} and h_{jp}, denote elements of the corresponding matrices, $\mathbf{V}$, $\mathbf{W}$ and $\mathbf{H}$, respectively. NMF can also be solved with other objective functions such as Frobenius norm or more general β-divergence, depending on the applications[24].

Notably, minimization of the generalized KL divergence is equivalent to maximize a likelihood function of a probabilistic NMF model[32,33]. Indeed, for a Poisson NMF model, v_{np} of the nth tumor and pth mutation catalog is assumed to be independently distributed, following a Poisson distribution with mean $\sum_{j=1}^{r} w_{nj} h_{jp}$. The log-likelihood of Poisson NMF model can be written as $\log\{\Pr(\mathbf{V}|\mathbf{WH})\} = -D_{KL}(\mathbf{V}|\mathbf{WH}) + C$ with a constant C. Therefore, minimizing generalized KL divergence $D_{KL}(\mathbf{V}|\mathbf{WH})$ is equivalent to maximizing the log-likelihood $\log\{\Pr(\mathbf{V}|\mathbf{WH})\}$. In addition, it could been shown that the multiplicative update algorithm[34], which is commonly used to minimize the generalized KL divergence, is equivalent to an expectation/conditional maximization (ECM) algorithm[30] for the Poisson NMF model (Supplementary Note 2). These two equivalences are used to develop SUITOR.

As mentioned above, NMF or equivalently the Poisson NMF model requires a given number of signatures r, which is unknown in practice; and SUITOR can select this number empirically. The steps of SUITOR are outlined as follows (with a schematic illustration in Fig.1): 1) the mutation catalog matrix is separated into the training and validation sets. The training set contains missing data held out as validation data; 2) the missing data are imputed in the initial step; 3) the ECM algorithm iteratively imputes the missing data in the expectation step (E-step) and estimates the signature contributions (of the **W** matrix) and profiles (of the **H** matrix) in the

conditional maximization steps (CM-steps) until the ECM algorithm allows $\mathbf{V} \approx \mathbf{WH}$ for a given number of signatures; 4) the missing data are imputed and compared to the validation data to calculate the prediction error in the validation set; 5) The above described steps are conducted for multiple candidate numbers of signatures and the one with the minimal prediction error will be chosen as the optimal number of signatures (corresponding to the red dot in the prediction error curve of validation set in Fig. 1).

There are three key contributions of SUITOR. First, SUITOR selects the number of signatures with the minimal prediction error, which prevents selecting too few or too many signatures (as model underfitting or overfitting). Although the prediction error in the training set is reduced with increasing signatures (as illustrated by the prediction error curve of the training set in Fig. 1), the prediction error in the validation set will decrease first (due to the model underfitting with insufficient signatures) and then inflate (due to the model overfitting with redundant signatures). This is the well-known bias-variance tradeoff for model complexity[35] measured by the number of signatures in the setting of mutational signature analysis. Second, the cross-validation scheme of SUITOR guarantees that the missing data pattern does not depend on the remaining or missing mutation counts in the training set. Hence, the missing data mechanism is missing completely at random (MCAR), which ensures that the estimated signature profiles and contributions would not be biased due to the missing data[36]. Third, the proposed ECM algorithm for SUITOR enjoys the convergence property, which guarantees the increase of the likelihood function over iterations until the ECM algorithm converges[30].

Evaluation of SUITOR in two *in vitro* studies

We assessed the performance of SUITOR in two experimental studies[2,5], for which the true number and profile of signatures were generated experimentally and validated *in vitro* (see details in Methods). The first study created endogenous mutational signatures through CRISPR-Cas9-mediated knockouts of DNA repair genes in an isogenic human cell line[5]. The second study generated exogenous mutational signatures in human-induced pluripotent stem cell (iPSC) lines exposed to environmental or therapeutic mutagens[2]. For both studies, we evaluated whether SUITOR could correctly select the number of signatures and recover the profiles of single base substitution signatures. We then compared SUITOR's performance with sigProfiler, signatureAnalyzer and signeR.

For the *in vitro* CRISPR-Cas9-mediated knockout study of the DNA repair gene *MSH6,* SUITOR correctly detected the background signature, which existed before the knockout of *MSH6* and *MSH6* knockout-induced signature (Fig. 2a) and recovered the corresponding signature profiles (Fig. 2b; Supplementary Table 1). SigProfiler, signatureAnalyzer and signeR correctly identified these two signatures as well (Supplementary Fig. 1; Supplementary Table 1). Next, we extracted signatures of knockout studies of six DNA repair genes (*CHEK2*, *NEIL1*, *NUDT1*, *POLB*, *POLE* and *POLM)*, which did not induce experimentally detectable signatures[5]. SUITOR and the other three methods correctly identified one background signature only without false detection of knockout-induced signatures (Supplementary Fig. 2). We conclude that in this *in vitro* study with at most two signatures, all four methods perform equally well. This is not the case when the number of signatures increases as shown below.

For the *in vitro* study of 79 exogenous mutagens, stable mutational signatures were experimentally identified for 28 mutagens. These 28 signatures are not distinct as some signatures are very similar to each other (e.g., benzo[a]pyrene (BaP) and benzo[a]pyrene-7,8-dihydrodiol-9,10-epoxide (BPDE), both of which are polycyclic aromatic hydrocarbons (PAHs), mutagens of tobacco smoke; Supplementary Fig. 3). Although all four methods could find the background mutational signature, SUITOR detected 9 additional signatures induced by mutagens (Figure 2c, 10 signatures, including one background signature and 9 mutagen-induced signatures), sigProfiler detected 4, signatureAnalyzer 8, and signeR 5 additional signatures (Figure 2d; Supplementary Fig. 4; Supplementary Table 2); the signature of 1,2-dimethylhydrazine (1,2-DMH) was detected by SUITOR and missed by the other methods. Besides detecting more true signatures, SUITOR also achieved the lowest prediction error (SUITOR:1292.5; sigProfiler:2295.4; signatureAnalyzer:2176.6; signeR:1585.1). This indicates that the signatures detected by SUITOR in the training set predicted most closely the mutation counts in the validation set. When we stratified the tumor subclones based on the signatures detected by at least one method, SUITOR and signatureAnalyzer were able to separate most subclones into distinct clusters, each corresponding to a unique mutagen exposure (Supplementary Fig. 5). In contrast, SigProfiler merged subclones exposed to BPDE and dibenz[a,h]anthracene diol-epoxide (DBADE); signeR mixed subclones exposed to N-ethyl-N-nitrosourea (ENU) and 1,2-DMH; and both SigProfiler and signeR mixed subclones exposed to 9-Nitrochrysense and aristolochic acid I (AAI).

We note that *in vitro* study of exogenous mutagens favors the method of signatureAnalyzer. SignatureAnalyzer implicitly assumes few signatures are present per sample and hence the

loadings of signatures are sparse, which holds here since each sample was treated with a single mutagen in this *in vitro* study of exogenous mutagens. In spite of that, SUITOR performed better than signatureAnalyzer. When the sparsity assumption does not hold, as demonstrated in *in silico* simulations and the PCAWG study described below, signatureAnalyzer would find more false-positive signatures than the other methods. In contrast, SUITOR, which does not rely on the sparsity assumption, is not susceptible to it.

***In silico* simulation studies**

We evaluated SUITOR and three other methods through additional *in silico* simulations. First, we evaluated the scenario when only random mutations occur. SUITOR identified one random signature in 20 of 20 replicates while other methods found many more signatures (as many as 96 signatures for signatureAnalyzer; Supplementary Table 3). Note that the random signature identified by SUITOR is similar to SBS3 (cosine similarity = 0.87), implying that SBS3 would possibly be caused by random mutations. Next, we considered a setting of one true signature, for which any additional signature found would be a false positive (details in Methods). SUITOR, sigProfiler and signeR correctly identified the single true signature (Supplementary Fig. 6) in 20 of 20 replicates; signatureAnalyzer found a false positive signature (Fig. 3a) in 17 of 20 replicates. Finally, we examined a setting of nine true signatures with varying signature contributions (details in Methods; Supplementary Fig. 7; Supplementary Table 4). All methods correctly identified six common signatures (SBS1, 2, 3, 5, 13, 18) in 20 of 20 replicates; SUITOR and signatureAnalyzer detected two rare signatures (SBS8,41) in more replicates than the other two methods (Fig. 3b). None of the methods identified the extremely rare and flat signature

SBS40 (present in one tumor only). Notably, all methods detected no other signatures besides the nine true signatures. Together, the simulation studies suggest that SUITOR is able to find both common and rare signatures while well controlling the rate of false positives.

Detection of pan-cancer mutational signatures

We tested the four methods in whole-genome sequencing (WGS) data of 2,540 tumors across 22 cancer types from the Pan-Cancer Analysis of Whole Genomes (PCAWG) study[1] (details in Methods).

First, we extracted *de novo* mutation signatures one cancer type at a time for eight cancer types, each with at least 100 tumors. Unlike in *in vitro* or *in silico* studies, the true signatures were unknown here. Nevertheless, we could evaluate if the signatures detected in part of the dataset predict mutation counts in the remaining part well. Specifically, the mutation catalog matrix is separated into training, validation and testing sets, the last of which is used to evaluate the performance of the selected number of signatures (details in Methods). Among the four methods, SUITOR clearly attained the smallest prediction errors across eight cancer types (Fig. 4a). Moreover, most signatures found by SUITOR were highly similar to the COSMIC signatures (with cosine similarity > 0.8, Fig. 4b) and frequently detected by the other methods (Fig. 4c). In contrast, signatureAnalyzer identified more *de novo* signatures, some of which were not matched to any COSMIC signatures (Fig. 4b).

Next, we extracted *de novo* signatures combining WGS data from 2,540 tumors (pan-cancer analysis). SUITOR found 22 signatures, eighteen of which could be matched to the COSMIC signatures (Fig. 4b). These signatures had the smallest prediction error (Fig. 4a), compared to signatures detected by the other methods: SUITOR 36,017; sigProfiler 255,111 (>7 times SUITOR's prediction error); signatureAnalyzer 402,409 (>11 times SUITOR's prediction error); and signeR 193,191 (>5 times SUITOR's prediction error). As expected, the signatures commonly found in multiple cancer types (e.g., SBS1, SBS2 and SBS13) could be identified when combining all cancer types together, while signatures specific to a single cancer type (e.g., SBS24 specific to liver cancer) were absent in the combined signature analysis (Fig. 4c). When we clustered 2,540 tumors based on signature contributions estimated by SUITOR, four clusters emerged in the t-SNE plot (Fig. 5a). Liver tumors formed a distinct cluster, possibly due to its specific signature SBS24 caused by aflatoxin exposure; the remaining clusters included: i) a subset of lymphomas; ii) the majority of kidney tumors; iii) all remaining tumors. Although the liver tumors were separated from the other tumors in the t-SNE plots also by the other methods (Fig. 5b-d), the separation of lymphomas and kidney tumors was less clear. For example, kidney tumors and lymphoma were mixed with other cancer types in the t-SNE plot from signatureAnalyzer.

External validation of breast cancer mutational signatures

We validated the nine PCAWG breast cancer signatures (based on 194 breast tumors) using an independent WGS set of 440 breast tumors of the Sanger breast cancer (BRCA) study from the same ethnicity[37]. SUITOR (Fig. 6a and Supplementary Table 5) and signeR (Supplementary Table

6) identified nine breast cancer signatures in PCAWG and validated eight in the Sanger BRCA study (with cosine similarity > 0.8); sigProfiler identified eight signatures and confirmed seven (Supplementary Table 7); signatureAnalyzer identified twelve and validated eight (Supplementary Table 8). Overall, six signatures (SBS1,2,8,13,17b and 18) were identified in both studies by all methods, while the flat featureless signature SBS5 could not be validated by any method.

Besides validating eight PCAWG breast cancer signatures, SUITOR found four additional signatures in the Sanger BRCA study (Fig. 6a and Supplementary Table 9). Two of them were highly similar to COSMIC signatures SBS26 and SBS30 (cosine similarity > 0.93) and were identified by the other three methods as well; the other two (similar to SBS3 and SBS6) were also detected by signatureAnalyzer and/or signeR. Similarly, three other methods found few more signatures as well (Supplementary Tables 10-12). These findings suggest that there likely exist additional signatures in the Sanger BRCA study that are missed in the PCAWG breast cancer study because of either the larger sample size or specific operative mutational processes (e.g., BRCA1 mutation carriers with signature SBS3) in Sanger BRCA study.

Finally, as an example of clinical utility of the signatures estimated by SUITOR, we stratified the 440 breast tumors of the Sanger BRCA study using the signature contributions. Four signature clusters were found; two dominant clusters (G3 and G4) included overall 430 tumors (Fig. 6b). Compared to the G4 subgroup, the G3 subgroup showed significantly higher contributions of the nine signatures (Supplementary Fig. 8; Supplementary Table 13) and significantly lower

contributions of the de novo signature A (similar to COSMIC signature SBS1 associated with aging). We found a number of clinical factors associated with the subgroups G3 and G4, including age at diagnosis and tumor grade (Supplementary Table 14). The singular most important associated factor was the molecular subtype: triple negative breast cancers were significantly enriched in the subgroup G3 (Fig. 6c; odds ratio = 25.1, P-value < 2.2×10^{-16}, two-sided Fisher's exact test).

Discussion

It's crucial to select the correct number of *de novo* mutational signatures for a cancer genomics study. Here we present SUITOR that selects the number of signatures through cross-validation to minimize the prediction error in the validation set. We have shown how SUITOR outperforms common existing methods most of the time. *In vitro* studies show that SUITOR is capable of retrieving the correct number and profiles of both endogenous and exogenous signatures, allowing the correct stratification of tumor subclones exposed to distinct mutagens. *In silico* simulation studies show that SUITOR can detect common signatures in all replicates and rare signatures (as low as 1%) in the majority of replicates. Applications to *in vivo* eight PCAWG cancer types show that SUITOR discovers signatures which achieve the lowest prediction errors in the testing sets. Most of these signatures are confirmed by other methods and matched to the COSMIC signatures. All except one signature found in PCAWG BRCA study were validated in the independent Sanger BRCA study. The contributions of signatures selected by SUITOR in the Sanger BRCA study are dominated by two clusters, driven by the molecular/histological subtypes.

Besides selecting the number of signatures of single base substitution, SUITOR could be used to select the number of signatures of other genomic alterations in tumors, including double base substitutions, small insertion and deletions (indels), and structure variations. In this paper, we used 10-fold cross validation, which is recommended as a good compromise for the bias-variance trade-off regarding the choice of k in k-fold cross validation[38,39]. In addition, we have tried 20-fold cross validation (i.e., 5% of data as validation data) for PCAWG studies, which led to the same number of signatures (results not shown).

In summary, SUITOR has shown to perform better than other commonly used methods in revealing mutational signatures, the "footprints" engraved in the cancer genomes by operative mutational processes with potentially important etiological or therapeutic implications.

Methods

Unsupervised cross-validation for mutational signature analysis

SUITOR aims to select the optimal number of signatures which minimizes the prediction error in the validation set through cross-validation. Here, we describe the steps to create the validation set and the related challenges.

For a K-fold cross-validation, we divide the catalog matrix $\mathbf{V}$ into K parts where the Poisson NMF model is fitted on $K-1$ parts as the training set, and the fitted model is validated on the remaining one part as the validation set. The cross-validation is carried out K times with each part served as a validation set once, using the balanced separation[40,41] detailed as follows. In the

k^{th} fold ($1 \leq k \leq K$) of a balanced separation, a set of mutation counts $\{v_{np} | p = (n \bmod 10) + (k-1) + aK, a = 1, 2, \dots.\}$ are held out for the nth tumor as validation data, where a is restricted such that $1 \leq p \leq 96$. For example, $(1, 11, \cdots, 91)^{st}$ mutation catalogs of the first tumor are held out in the 1st fold, $(2, 12, \cdots, 92)^{nd}$ mutation catalogs in the 2nd fold and so on. Note that the balanced separation keeps equal number of retained mutation catalogs for each tumor in the training set, which is computationally more stable than randomly splitting **V** into the training and validation sets. The latter may randomly remove a large number of mutation catalogs for a tumor.

As validation data are hold out, missing data emerge in the training set, the reason for which existing methods of NMF fail. To address this challenge, we extended the Poisson NMF model and propose an expectation/conditional maximization (ECM) algorithm to incorporate the missing data.

Expectation/conditional maximization (ECM) algorithm of SUITOR

Let $\mathcal{S}$ be the set of indices of mutation catalog matrix **V** such that $\mathcal{S} = \{(n, p) | 1 \leq n \leq \text{N and } 1 \leq p \leq 96\}$. For a K-fold cross-validation, $\mathcal{S}$ would be divided into K disjoint sets $\mathcal{S}_1, \cdots, \mathcal{S}_K$. For the kth fold, $\mathbf{V}_k^L = \{v_{np} | (n, p) \in \mathcal{S}_k\}$ denotes the validation set and $\mathbf{V}_k^T = \{v_{np} | (n, p) \in \mathcal{S} \backslash \mathcal{S}_k\}$ the training set, where $\mathcal{S} \backslash \mathcal{S}_k$ represents the indices of **V** excluding ones in $\mathcal{S}_k$. Mutation counts in $\mathcal{S}_k$ will be removed from **V** and denote as missing data $\mathbf{M}_k = \{m_{np} | (n, p) \in \mathcal{S}_k\}$. The v_{np} in $\mathbf{V}_k^T$ and m_{np} in $\mathbf{M}_k$ are assumed to be independently distributed as a Poisson distribution with the mean $\sum_{j=1}^{r} w_{nj} h_{jp}$. By the scheme of balanced separation, $\mathbf{V}_k^L$ is missing completely at random (MCAR), since $\mathbf{V}_k^L$ is removed from **V**, independent of values of

$\mathbf{V}_k^T$ and $\mathbf{V}_k^L$. MCAR enables us to propose an ECM algorithm to incorporate missing data and obtain unbiased estimates of $\mathbf{W}$ and $\mathbf{H}$ (ref[36]).

Next, we outline the ECM algorithm in the following iterative steps (details in Supplementary Note 3).

1. Initial step: choose initial values of $\mathbf{M}_k$ and set initial parameters $\mathbf{W}^{\mathbf{0}}$ and $\mathbf{H}^{\mathbf{0}}$.
2. E-step: given the observed data $\mathbf{V}_k^T$ and the parameters $\mathbf{W}^{\boldsymbol{t}}$ and $\mathbf{H}^{\boldsymbol{t}}$ of the previous step t, the ECM algorithm calculates the conditional expectation of complete likelihood

$$Q(\mathbf{W}, \mathbf{H}|\mathbf{W}^{\boldsymbol{t}}, \mathbf{H}^{\boldsymbol{t}}) = E[\log\{\Pr(\mathbf{V}_k^T, \mathbf{M}_k|\mathbf{W}\mathbf{H})\}\,|\mathbf{W}^{\boldsymbol{t}}, \mathbf{H}^{\boldsymbol{t}}\}] = -D_{KL}(\mathbf{V}^*|\mathbf{W}\mathbf{H}) + C^*,$$

   where C^* is a constant independent of $\mathbf{W}$ and $\mathbf{H}$, v_{np}^* the elements of $\mathbf{V}^*$ as $v_{np}^* = v_{np}$ for $(n, p) \in \mathcal{S} \backslash \mathcal{S}_k$ and $v_{np}^* = E[m_{np}] = \sum_{j=1}^{r} w_{nj}^t h_{jp}^t$ for $(n, p) \in \mathcal{S}_k$.
3. CM1-step: update parameters $\mathbf{W}^{\boldsymbol{t+1}}$ by maximizing $Q(\mathbf{W}, \mathbf{H}^{\boldsymbol{t}}|\mathbf{W}^{\boldsymbol{t}}, \mathbf{H}^{\boldsymbol{t}})$ with respect to $\mathbf{W}$.
4. CM2-step: update parameters $\mathbf{H}^{\boldsymbol{t+1}}$ by maximizing $Q(\mathbf{W}^{\boldsymbol{t+1}}, \mathbf{H}\big|\mathbf{W}^{\boldsymbol{t}}, \mathbf{H}^{\boldsymbol{t}})$ with respect to $\mathbf{H}$.
5. Iterate steps 2 to 4 until convergence.

In the initial step, we use the median of mutation counts in $\mathbf{V}_k^T$ per each mutation catalog as initial values for $\mathbf{M}_k$; other more complicated methods of specifying initial values, such as nearest neighbors, lead to similar results (results not shown). We are aware that the ECM algorithm possibly converges to a local saddle point. To overcome it, we try 300 random initial values $\mathbf{W}^{\mathbf{0}}$ and $\mathbf{H}^{\mathbf{0}}$, which leads to 300 pairs of $\widehat{\boldsymbol{W}}_i$ and $\widehat{\boldsymbol{H}}_i$, the estimates of $\mathbf{W}$ and $\mathbf{H}$ for the ith

initial value, $i = 1, 2, \dots, 300$. The final reported $\widehat{\mathbf{W}}$ and $\widehat{\mathbf{H}}$ are the $\widehat{\boldsymbol{W}}_i$ and $\widehat{\boldsymbol{H}}_i$ which maximize the $\log\{\Pr(\mathbf{V}_k^T | \widehat{\boldsymbol{W}}_l, \widehat{\boldsymbol{H}}_l)\}$ among all $\widehat{\boldsymbol{W}}_i's$ and $\widehat{\boldsymbol{H}}_i's$.

Selecting number of signatures by SUITOR

For a given number of signatures r, we first evaluate the prediction error, i.e., the disparity between the observed validation data $\mathbf{V}_k^L$ and the predicted ones $\widehat{\boldsymbol{M}}_k = \mathbf{E}[\mathbf{M}_k] = \left\{ \left(\widehat{\mathbf{W}}\widehat{\mathbf{H}}\right)_{np} \middle| (n,p) \in \mathcal{S}_k \right\}$, for the kth fold, $k = 1,2, \dots, K$,

$$ERR_{r,k} \equiv -\log\{\Pr(\mathbf{V}_k^L | \widehat{\boldsymbol{M}}_k)\} = D_{KL}(\mathbf{V}_k^L | \widehat{\boldsymbol{M}}_k) - C^*.$$

We then evaluate overall prediction error, $ERR_r = \sum_{k=1}^{K} ERR_{r,k}$, across K folds. Since the term C^* is unrelated to $\widehat{\mathbf{W}}$ and $\widehat{\mathbf{H}}$, it is dropped. Finally, we select the number of signatures r^* which minimizes ERR_r over a range of numbers of signatures $1 \leq r \leq R$.

Extracting signature profiles and estimating contributions of signatures

Once the optimal number of signatures r^* is determined by SUITOR, we extract mutational signature profiles $\mathbf{W}$ and estimating contributions $\mathbf{H}$, via maximizing $\log\{\Pr(\mathbf{V}|\mathbf{WH})\}$ with the fixed rank r^*. Similar to the ECM algorithm in SUITOR, we evaluate multiple initial values and use $\widehat{\mathbf{W}}$ and $\widehat{\mathbf{H}}$ which maximizes $\log\{\Pr(\mathbf{V} | \widehat{\mathbf{W}}\widehat{\mathbf{H}})\}$ to relieve local optima problem.

***In vitro* studies**

The datasets of two *in vitro* studies were downloaded from http://medgen.medschl.cam.ac.uk/serena-nik-zainal/. The details of study design and implementation were described previously[2,5]. In these *in vitro* studies, the endogenous and

exogenous mutational signatures were experimentally generated *in vitro* and hence the true number of signatures and profiles are known.

We created the mutation catalog matrix for both studies and applied SUITOR, sigProfiler, signatureAnalyzer and signeR. We chose sigProfiler and signatureAnalyzer, since they have been applied to a number of studies[7,8,18,42-44], and signeR[17] because it utilizes Bayesian information criterion (BIC), a popular model selection criterion for supervised learning. For SUITOR, we used 10-fold cross-validation with 90% of counts in mutation catalog matrix as the training set and the remaining 10% as the validation set. In contrast, the whole mutation catalog matrix **V** was analyzed by sigProfiler, signatureAnalyzer and signeR, respectively under the default setting.

The first study induced endogenous mutational signatures by CRISPR-Cas9-mediated knockouts of DNA repair genes in an isogenic human cell line. First, we focused on the *MSH6* knockout-induced single base substitution signature, which is characterized by C>T and T>C single base substitutions (~148 substitutions per cell division). We evaluated whether the four methods were able to retrieve the background signature and the *MSH6* knockout-induced signatures. Next, we analyzed the gene-knockout studies with no induced signatures (for genes *CHK2, NEIL1, NUDT1, POLB, POLE* and *POLM*), to evaluate whether the four methods would find false positive signatures in addition to the background signature.

In the second study, exogenous mutational signatures were created by environmental or therapeutic mutagens. We selected 324 subclones (including 35 control subclones) of human-

induced pluripotent stem cell (iPSC) lines, for which the mutations were measured by whole-genome sequencing (WGS). While controls are not exposed to mutagens, each subclone is exposed to one of 79 mutagens, including simulated solar radiation (SSR), dibenzo[a,l]pyrene (DBP) and alkylating agent therapy temozolomide (TMZ). SSR recapitulates the UV-associated signatures and DBP is a potent carcinogen of the polycyclic aromatic hydrocarbons (PAHs) produced when coal, crude oil, or gasoline is burned. For each method, we checked if the correct number of signatures were attained with its impacts on the downstream analyses. Specifically, we investigated whether the retrieved *de novo* signature profiles were highly similar to the true signature profiles. We further explored if signature contributes could separate subclones exposed to the distinctive mutagens, visualized by t-distributed stochastic neighbor embedding (t-SNE[45]).

In silico **simulation design with random mutations**

We simulated mutation catalog matrix for 300 tumors directly from the Poisson distribution with the mean lambda, which in turn was drawn from a uniform distribution and varied from 20 to 160. We repeated this simulation 20 times and analyzed the simulated catalog matrices by SUITOR, sigProfiler, signatureAnalyzer and signeR.

In silico **simulation design with one signature**

We simulated a mutation catalog matrix for 500 tumors and analyzed it by the four methods, each repeated 20 times. We used the signature profile of SBS8 as the true signature profile for **H** and generated the contribution vector **W** from a uniform distribution within the range [20000, 40000]. Then the mutation catalog **V** was generated by a Poisson distribution with mean **WH**.

In silico simulation design with nine signatures

We simulated signatures mimic to the ones observed in the Pan-Cancer Analysis of Whole Genomes (PCAWG) breast cancer study[1]. The nine signatures identified by SUITOR show various signature contributions and signature profiles; some signatures contribute to all tumors (e.g., SBS1 and SBS5, present in 100% of tumors; Supplementary Table 4) while others contribute to a few tumors (e.g., SBS41, present in 6% of tumors) and even to one (SBS40) or two tumors (SBS8); some signature profiles are spiky (e.g., SBS1 and SBS2/13; Supplementary Fig. 5) while others are relatively flat (e.g., SBS5).

Specifically, we generated mutation catalog matrices similar to $\mathbf{V}^{\mathrm{BR}}$, the mutation catalog matrix of PCAWG breast cancer study. $\mathbf{V}^{\mathrm{BR}}$ is approximated by $\mathbf{W}^{\mathrm{BR}}\mathbf{H}$, for which $\mathbf{H}$ contains the COSMIC signature profiles and $\mathbf{W}^{\mathrm{BR}}$ is the corresponding signature contribution matrix (downloaded from https://www.synapse.org/#!Synapse:syn11738669). We removed signatures with zero contributions to all tumors and chose 9 signatures to compose the signature profile matrix $\mathbf{H}$ of size 9×96. With $\mathbf{H}$ fixed, we took bootstrap samples $\boldsymbol{W}_{\boldsymbol{boot}}^{\boldsymbol{BR}}$ from each column of matrix $\mathbf{W}^{\mathrm{BR}}$, and generated $\boldsymbol{V}_{\boldsymbol{boot}}^{\boldsymbol{BR}}$ which follows a Poisson distribution with mean $\boldsymbol{W}_{\boldsymbol{boot}}^{\boldsymbol{BR}}\,\mathbf{H}$. Due to the dependencies between SBS1 and SBS5 as well as between SBS2 and SBS13, their contributions are resampled jointly while the contributions of other signatures are sampled individually. We simulated 20 mutation catalog matrices for 200 tumors, and each was analyzed by four methods respectively.

In vivo human cancer genomics studies

We analyzed 2, 540 tumors across 22 cancer types of PCAWG[1], including 321 tumors of hepatocellular carcinoma, 286 tumors of prostate adenocarcinoma, 237 tumors of pancreatic adenocarcinoma, 194 tumors of breast adenocarcinoma, 146 tumors of central nervous system medulloblastoma, 143 tumors of renal cell carcinoma,112 tumors of ovary adenocarcinoma and 100 cases of B-cell non-Hodgkin lymphoma. Other cancer types have less than 100 tumors per cancer type. The tumors were whole genome sequenced and datasets of the somatic mutation calls were downloaded from https://www.synapse.org/#!Synapse:syn11726620. The hypermutator tumors with mutation burden more than 10 mutations/Mb were excluded[46]. For each cancer type, we applied SUITOR, sigProfiler, signatureAnalyzer and signeR to select the number of signatures and estimate signature contributions and profiles.

To compare the prediction errors, we split the mutation catalog matrix into a training set (90% of counts in the mutation catalog matrix), a validation set (5%) and a testing set (5%). For SUITOR, the training set was used to fit the probabilistic NMF model with multiple numbers of signatures and the validation set to select the number of signatures. The other methods used both the training and validation sets to select the number of signatures. Next, we compared the prediction errors of selected signatures by each method on the testing set. For sigProfiler, signatureAnalyzer and signeR, we imputed missing training data by medians of available mutation counts per each mutational catalog, applied each method, predicted the testing data and calculated the prediction error as SUITOR did. For SUITOR, it could handle missing data and predict the testing data by the ECM algorithm.

Sanger whole genome sequencing breast cancer study

The Sanger whole genome breast cancer (BRCA) study sequenced 560 breast tumors. The somatic mutation calls files were downloaded from https://medgen.medschl.cam.ac.uk/serena-nik-zainal/. Among 560 breast tumors, 110 tumors were included in PCAWG and hence excluded from this validation study. Ten hypermutator tumors were also excluded. We applied SUITOR, sigProfiler, signatureAnalyzer and signeR to a) select the number of signatures and estimate signature contributions and profiles; b) compare the signatures with ones detected in the PCAWG breast cancer study; and c) investigate if additional signatures are found in Sanger whole genome breast cancer study. In addition, we stratified the tumors based on mutation contributions and associated the signature clusters with epidemiological or clinical characteristics.

Software webpages:

SUITOR: https://github.com/binzhulab/SUITOR

sigProfiler: v1.0.6 is installed and used by following instructions in https://github.com/AlexandrovLab/SigProfilerExtractor.

signatureAnalyzer: version information is not available but is installed by following instructions in https://github.com/broadinstitute/getzlab-SignatureAnalyzer.

signeR: v1.12.0 is downloaded from https://bioconductor.org/packages/release/bioc/html/signeR.html.

sigProfiler and signatureAnalyzer are used in python version 3.7.5 while SUITOR and signeR are used in R version 3.6.3. Signature profiles plot is drawn based on R package MutationalPatterns from https://bioconductor.org/packages/release/bioc/html/MutationalPatterns.html with slight modification to add graphical parameters.

Acknowledgment

This research was supported by the Intramural Research Program of the National Institutes of Health, National Cancer Institute, Division of Cancer Epidemiology and Genetics (DCEG). This study utilized the high-performance computational capabilities of the Biowulf Linux cluster at the National Institutes of Health, Bethesda, MD: https://biowulf.nih.gov. We would like to thank Bill Wheeler (Information Management Services) for computation support and Dr. Paul Albert (Biostatistics Branch of DCEG) and Dr. Ludmil Alexandrov (University of California San Diego) for helpful comments. Conflict of Interest: None declared.

Reference:

1. Alexandrov, L.B. *et al.* The repertoire of mutational signatures in human cancer. *Nature* **578**, 94-101 (2020).
2. Kucab, J.E. *et al.* A Compendium of Mutational Signatures of Environmental Agents. *Cell* **177**, 821-836 e16 (2019).
3. Alexandrov, L.B. *et al.* Mutational signatures associated with tobacco smoking in human cancer. *Science* **354**, 618-622 (2016).
4. Letouze, E. *et al.* Mutational signatures reveal the dynamic interplay of risk factors and cellular processes during liver tumorigenesis. *Nat Commun* **8**, 1315 (2017).

5. Zou, X. *et al.* Validating the concept of mutational signatures with isogenic cell models. *Nat Commun* **9**, 1744 (2018).
6. Polak, P. *et al.* A mutational signature reveals alterations underlying deficient homologous recombination repair in breast cancer. *Nat Genet* **49**, 1476-1486 (2017).
7. Haradhvala, N.J. *et al.* Distinct mutational signatures characterize concurrent loss of polymerase proofreading and mismatch repair. *Nat Commun* **9**, 1746 (2018).
8. Petljak, M. *et al.* Characterizing Mutational Signatures in Human Cancer Cell Lines Reveals Episodic APOBEC Mutagenesis. *Cell* **176**, 1282-1294 e20 (2019).
9. Drost, J. *et al.* Use of CRISPR-modified human stem cell organoids to study the origin of mutational signatures in cancer. *Science* **358**, 234-238 (2017).
10. Secrier, M. *et al.* Mutational signatures in esophageal adenocarcinoma define etiologically distinct subgroups with therapeutic relevance. *Nat Genet* **48**, 1131-41 (2016).
11. Staaf, J. *et al.* Whole-genome sequencing of triple-negative breast cancers in a population-based clinical study. *Nat Med* **25**, 1526-1533 (2019).
12. Connor, A.A. *et al.* Association of Distinct Mutational Signatures With Correlates of Increased Immune Activity in Pancreatic Ductal Adenocarcinoma. *JAMA Oncol* **3**, 774-783 (2017).
13. Li, X. *et al.* Distinct Subtypes of Gastric Cancer Defined by Molecular Characterization Include Novel Mutational Signatures with Prognostic Capability. *Cancer Res* **76**, 1724-32 (2016).
14. Gehring, J.S., Fischer, B., Lawrence, M. & Huber, W. SomaticSignatures: inferring mutational signatures from single-nucleotide variants. *Bioinformatics* **31**, 3673-5 (2015).
15. Alexandrov, L.B., Nik-Zainal, S., Wedge, D.C., Campbell, P.J. & Stratton, M.R. Deciphering signatures of mutational processes operative in human cancer. *Cell Rep* **3**, 246-59 (2013).
16. Fischer, A., Illingworth, C.J., Campbell, P.J. & Mustonen, V. EMu: probabilistic inference of mutational processes and their localization in the cancer genome. *Genome Biol* **14**, R39 (2013).
17. Rosales, R.A., Drummond, R.D., Valieris, R., Dias-Neto, E. & da Silva, I.T. signeR: an empirical Bayesian approach to mutational signature discovery. *Bioinformatics* **33**, 8-16 (2017).
18. Kim, J. *et al.* Somatic ERCC2 mutations are associated with a distinct genomic signature in urothelial tumors. *Nat Genet* **48**, 600-606 (2016).
19. Rosenthal, R., McGranahan, N., Herrero, J., Taylor, B.S. & Swanton, C. DeconstructSigs: delineating mutational processes in single tumors distinguishes DNA repair deficiencies and patterns of carcinoma evolution. *Genome Biol* **17**, 31 (2016).
20. Huang, X., Wojtowicz, D. & Przytycka, T.M. Detecting presence of mutational signatures in cancer with confidence. *Bioinformatics* **34**, 330-337 (2018).
21. Maura, F. *et al.* A practical guide for mutational signature analysis in hematological malignancies. *Nat Commun* **10**, 2969 (2019).
22. Degasperi, A. *et al.* A practical framework and online tool for mutational signature analyses show inter-tissue variation and driver dependencies. *Nat Cancer* **1**, 249-263 (2020).
23. Schwarz, G. Estimating the dimension of a model. *The annals of statistics* **6**, 461-464 (1978).
24. Tan, V.Y. & Févotte, C. Automatic relevance determination in nonnegative matrix factorization with the β-divergence. *IEEE Trans Pattern Anal Mach Intell* **35**, 1592-605 (2013).
25. Zhang, Y. & Yang, Y. Cross-validation for selecting a model selection procedure. *Journal of Econometrics* **187**, 95-112 (2015).

26. Arlot, S. & Celisse, A. A survey of cross-validation procedures for model selection. *Statistics surveys* **4**, 40-79 (2010).
27. Dyrskjot, L. *et al.* Identifying distinct classes of bladder carcinoma using microarrays. *Nat Genet* **33**, 90-6 (2003).
28. Byrne, R.P. *et al.* Dutch population structure across space, time and GWAS design. *Nat Commun* **11**, 4556 (2020).
29. Yu, J. *et al.* Lymph node metastasis prediction of papillary thyroid carcinoma based on transfer learning radiomics. *Nat Commun* **11**, 4807 (2020).
30. Meng, X.-L. & Rubin, D.B. Maximum Likelihood Estimation via the ECM Algorithm: A General Framework. *Biometrika* **80**, 267-278 (1993).
31. Févotte, C. & Cemgil, A.T. Nonnegative matrix factorizations as probabilistic inference in composite models. in *2009 17th European Signal Processing Conference* 1913-1917 (2009).
32. Gaussier, E. & Goutte, C. Relation between PLSA and NMF and implications. in *Proceedings of the 28th annual international ACM SIGIR conference on Research and development in information retrieval* 601–602 (Association for Computing Machinery, Salvador, Brazil, 2005).
33. Ding, C., Li, T. & Peng, W. Nonnegative matrix factorization and probabilistic latent semantic indexing: equivalence, chi-square statistic, and a hybrid method. in *Proceedings of the 21st national conference on Artificial intelligence - Volume 1* 342–347 (AAAI Press, Boston, Massachusetts, 2006).
34. Lee, D.D. & Seung, H.S. Learning the parts of objects by non-negative matrix factorization. *Nature* **401**, 788-91 (1999).
35. Friedman, J., Hastie, T. & Tibshirani, R. *The elements of statistical learning*, (Springer series in statistics New York, 2001).
36. Little, R.J. & Rubin, D.B. *Statistical analysis with missing data*, (John Wiley & Sons, 2019).
37. Nik-Zainal, S. *et al.* Landscape of somatic mutations in 560 breast cancer whole-genome sequences. *Nature* **534**, 47-54 (2016).
38. Kohavi, R. A study of cross-validation and bootstrap for accuracy estimation and model selection. in *Proceedings of the 14th international joint conference on Artificial intelligence - Volume 2* 1137–1143 (Morgan Kaufmann Publishers Inc., Montreal, Quebec, Canada, 1995).
39. Breiman, L. & Spector, P. Submodel Selection and Evaluation in Regression. The X-Random Case. *International Statistical Review / Revue Internationale de Statistique* **60**, 291-319 (1992).
40. Bro, R., Kjeldahl, K., Smilde, A.K. & Kiers, H.A.L. Cross-validation of component models: A critical look at current methods. *Analytical and Bioanalytical Chemistry* **390**, 1241-1251 (2008).
41. Wold, S. Cross-Validatory Estimation of the Number of Components in Factor and Principal Components Models. *Technometrics* **20**, 397-405 (1978).
42. Xue, R. *et al.* Genomic and Transcriptomic Profiling of Combined Hepatocellular and Intrahepatic Cholangiocarcinoma Reveals Distinct Molecular Subtypes. *Cancer Cell* **35**, 932-947 e8 (2019).
43. Rose Li, Y. *et al.* Mutational signatures in tumours induced by high and low energy radiation in Trp53 deficient mice. *Nat Commun* **11**, 394 (2020).
44. Kasar, S. *et al.* Whole-genome sequencing reveals activation-induced cytidine deaminase signatures during indolent chronic lymphocytic leukaemia evolution. *Nat Commun* **6**, 8866 (2015).
45. Maaten, L.v.d. & Hinton, G. Visualizing data using t-SNE. *Journal of machine learning research* **9**, 2579-2605 (2008).

46. Campbell, B.B. *et al.* Comprehensive Analysis of Hypermutation in Human Cancer. *Cell* **171**, 1042-1056 e10 (2017).

Figure legends:

Fig.1 A schematic overview of SUITOR. This schematic diagram illustrates how SUITOR selects the number of *de novo* mutational signatures. Details are given in Results. Each row of the mutation catalog matrix $\boldsymbol{V}$ represents a tumor, each column a mutation catalog. The bottom left shows prediction error curves in the training set (blue) and validation set (red), which are manually drawn for the illustration purpose, with the red dot representing the minimal prediction error in the validation set. ECM algorithm: expectation/conditional maximization algorithm; CM-steps: conditional maximization steps; E-step: expectation step; $\mathbf{W}$: signature contribution matrix of size $\mathrm{N} \times r$, N the number of tumor and r the number of signatures; $\mathbf{H}$: signature profile matrix of size $r \times 96$; $Q(\boldsymbol{W}, \boldsymbol{H}|\boldsymbol{W}^{\boldsymbol{t}}, \boldsymbol{H}^{\boldsymbol{t}})$: conditional expectation of complete likelihood of $\mathbf{W}$ and $\mathbf{H}$ at the $(t+1)$-th step, given estimated $\boldsymbol{W}^{\boldsymbol{t}}$ and $\boldsymbol{H}^{\boldsymbol{t}}$ in the t-th step of ECM algorithm.

Fig.2 *In vitro* evaluation of SUITOR and other methods. a) Prediction errors of SUITOR for the training and validation sets of *in vitro* knockout study of DNA repair gene *MSH6*. The red dot denotes the number of signatures with the minimal prediction error in the validation set. b) Profiles of single base substitution signature estimated by SUITOR in *MSH6* gene knockout study. The x-axis indicates the 5’ and 3’ nucleotides for each substitution type (e.g., T[C>A]C, cytosine to adenine substitution with 5’ thymine and 3’ cytosine). The top panel: the true and estimated background signatures (cosine similarity=0.991; cosine similarity=1 suggests two profiles being identical.); the bottom panel: the true and estimated *MSH6* knockout-induced

signatures (cosine similarity=0.997). c) Prediction errors by SUITOR for the training and validation sets of *in vitro* study of environmental or therapeutic mutagens. The red dot indicates the minimal prediction error in the validation set achieved by ten signatures, including one background signature and nine mutagen-induced signatures. d) Signatures discovered from the *in vitro* study of environmental or therapeutic mutagens by four methods. Methods which could identify a given mutagen are enclosed in paratheses. ENU: N-ethyl-N-nitrosourea; 6-Nitrochrysene+S9: 6-Nitrochrysene mixed with S9 rodent liver-derived metabolic enzyme; DES: diethyl sulfate; SSR: simulated solar radiation; AAI: aristolochic acid I; 1,2-DMH+S9: 1,2-dimethylhydrazine mixed with S9 rodent liver-derived metabolic enzyme; BPDE: benzo[a]pyrene-7,8-dihydrodiol-9,10-epoxide; DBADE: dibenz[a,h]anthracene diol-epoxide.

Fig.3 *In silico* evaluation of SUITOR and other methods. a) The signature profiles of the true mutational signature and the ones discovered by each method for a replicate. b) The number of replicates in which a given signature will be detected by each method; the rare signatures SBS8 and SBS41 were discovered in 12, 6, 14 and 6 replicates, and 17, 7, 19 and 11 replicates over total 20 replicates by SUITOR, sigProfiler, signatureAnalyzer and signeR, respectively.

Fig.4 Mutational signature results of eight cancer studies of PCAWG. a) The prediction errors of SUITOR, sigProfiler, signatureAnalyzer and signeR for eight cancer types (scale on the left-side Y axis) and for 2,540 tumors across 22 cancer types together, namely Pan-Cancer (scale on the right-side Y axis). SD: standard deviation. b) The number of signatures identified by each method. The shaded bars and the numbers above indicate the number of signatures whose

profiles could be matched to COSMIC profiles (with cosine similarity > 0.8). c) The cosine similarities between *de novo* signatures and COSMIC signatures. Only the matched pairs are shown (with cosine similarity > 0.8). The higher the cosine similarity the better match to a COSMIC signature profile. The cosine similarity equivalent to one denotes a perfect match.

Fig.5 The t-SNE visualization of clustering patterns for 2,540 tumors across 22 cancer types based on signature contributions. Signature contributions are estimated by four methods: (a) SUITOR, (b) sigProfiler, (c) signatureAnalyzer and (d) signeR. Each dot represents a tumor and is colored by the cancer type.

Fig.6 The results of Sanger breast cancer study by SUITOR. a) The heatmap of cosine similarity between *de novo* signatures detected in the PCAWG breast cancer (BRCA) study and *de novo* signatures in the Sanger BRCA study. The signatures of the Sanger BRCA study are annotated by COSMIC signatures (if cosine similarities > 0.8) among parentheses. For example, DNSigA (SBS1) refers to *de novo* signature A (DNSigA) being annotated by COSMIC signature SBS1. b) The heatmap of signature contributions estimated by SUITOR with hierarchical clustering in the Sanger BRCA study. The number of tumors included in each signature cluster is shown among parentheses on the top of the heatmap. Q1, Q2, Q3: the 1st, 2nd and 3rd quantiles of signature contributions. c) The t-SNE visualization of tumors in G3 and G4 signature clusters, color-coded by signature clusters and molecular subtypes. TNBC: triple negative breast cancer.

Figure
Click here to access/download;Figure;MainFigures.pdf

Figure 1

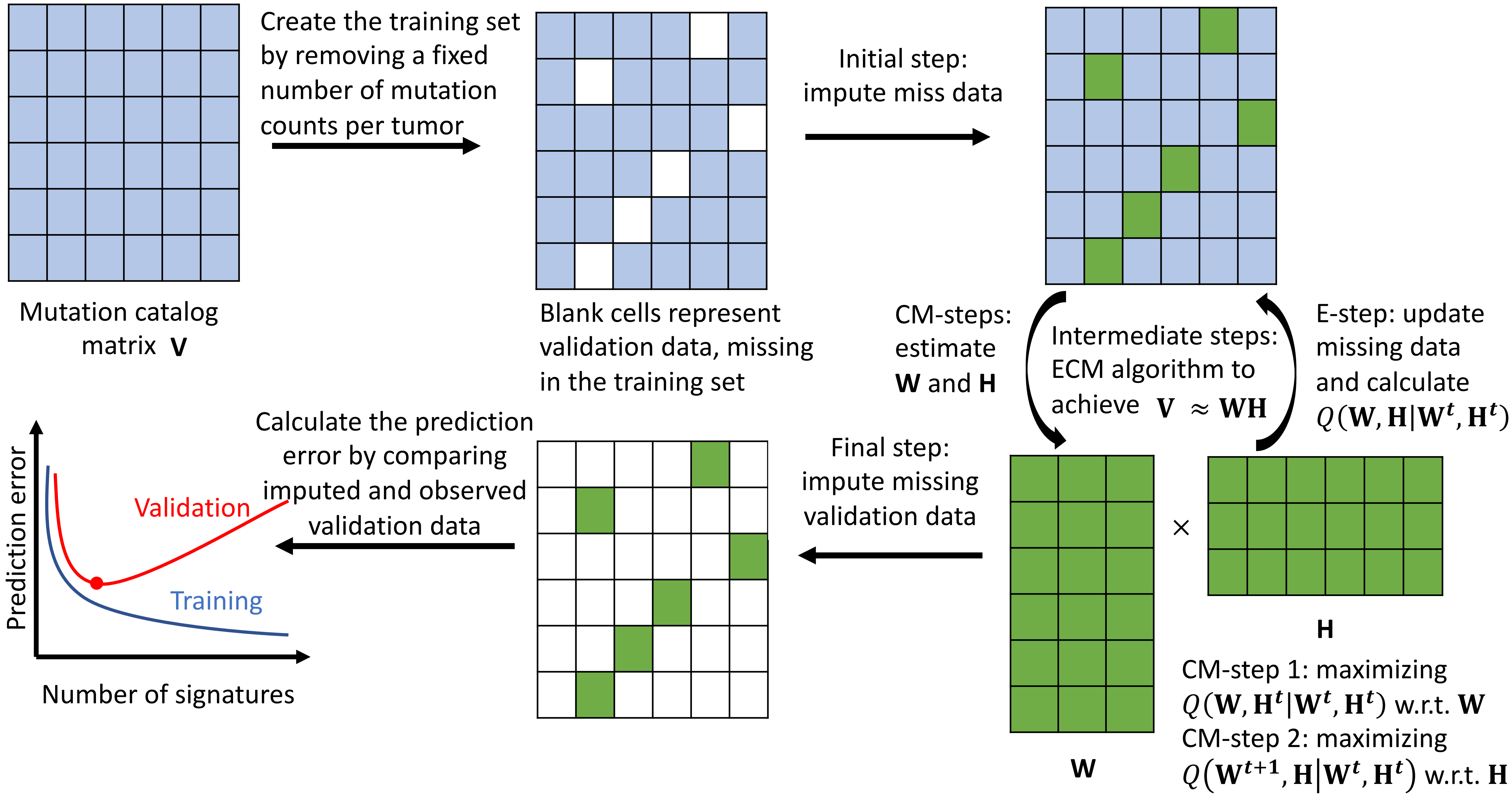

Figure 2

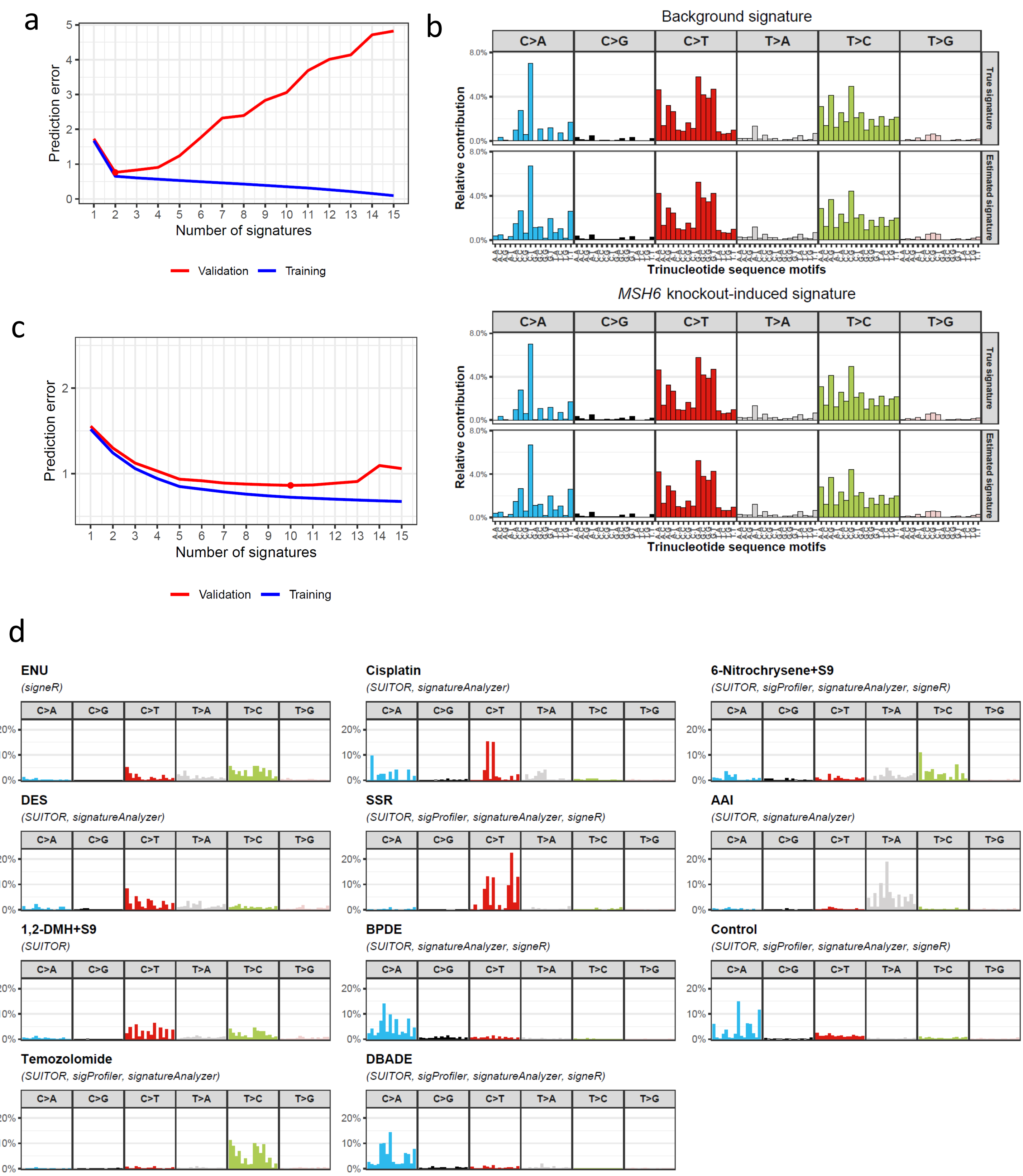

a
Prediction error
Number of signatures
Validation
Training
b
Background signature
C>A
C>G
C>T
T>A
T>C
T>G
True signature
Estimated signature
Relative contribution
Trinucleotide sequence motifs
MSH6 knockout-induced signature
c
Prediction error
Number of signatures
Validation
Training
d
ENU
(signeR)
Cisplatin
(SUITOR, signatureAnalyzer)
6-Nitrochrysene+S9
(SUITOR, sigProfiler, signatureAnalyzer, signeR)
DES
(SUITOR, signatureAnalyzer)
SSR
(SUITOR, sigProfiler, signatureAnalyzer, signeR)
AAI
(SUITOR, signatureAnalyzer)
1,2-DMH+S9
(SUITOR)
BPDE
(SUITOR, signatureAnalyzer, signeR)
Control
(SUITOR, sigProfiler, signatureAnalyzer, signeR)
Temozolomide
(SUITOR, sigProfiler, signatureAnalyzer)
DBADE
(SUITOR, sigProfiler, signatureAnalyzer, signeR)

Figure 3

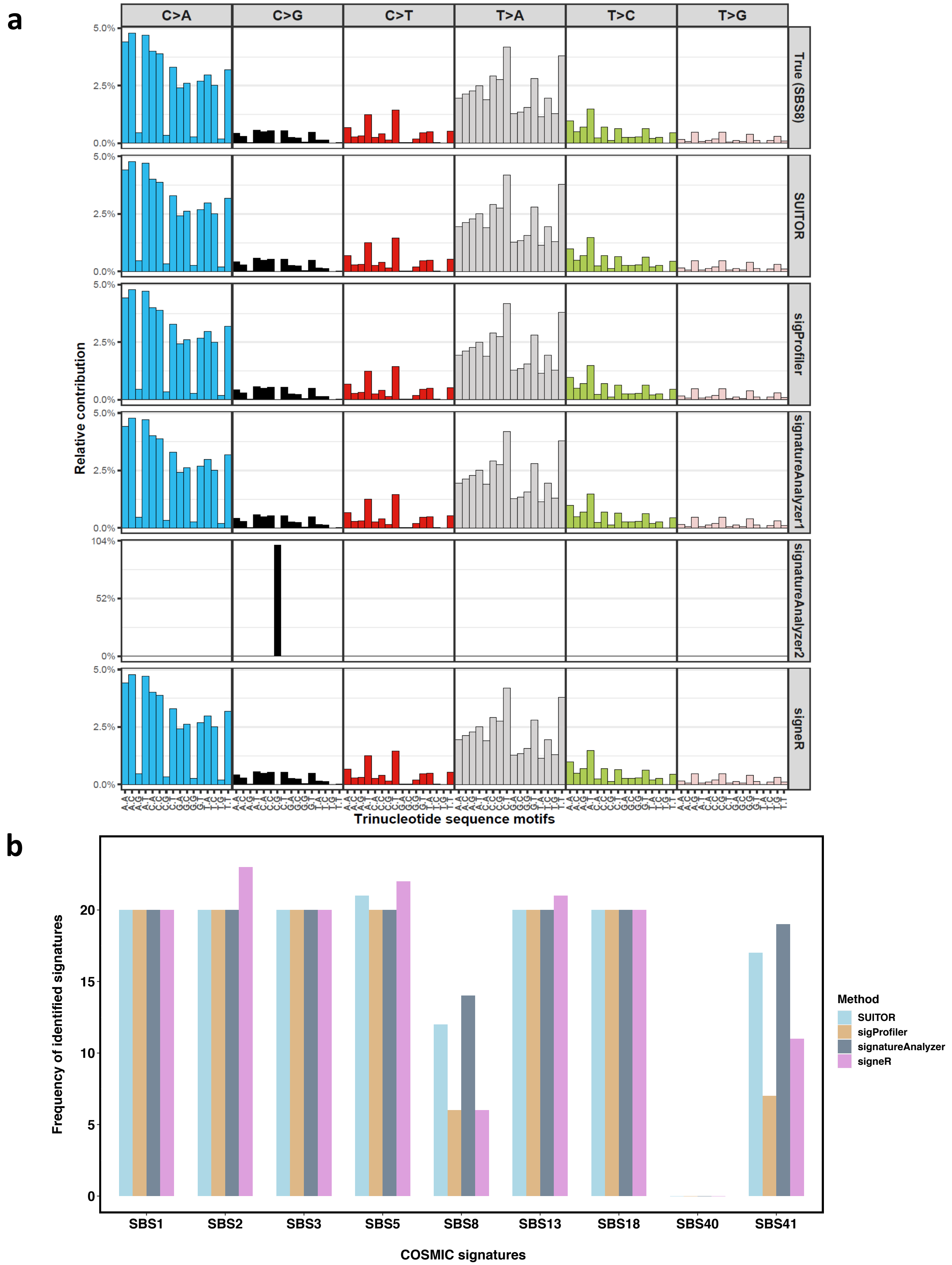
a
C>A
C>G
C>T
T>A
T>C
T>G
True (SBS8)
SUITOR
sigProfiler
signatureAnalyzer1
signatureAnalyzer2
signeR
Relative contribution
5.0%
2.5%
0.0%
104%
52%
0%
Trinucleotide sequence motifs
b
Frequency of identified signatures
0
5
10
15
20
SBS1
SBS2
SBS3
SBS5
SBS8
SBS13
SBS18
SBS40
SBS41
COSMIC signatures
Method
SUITOR
sigProfiler
signatureAnalyzer
signeR

Figure 4

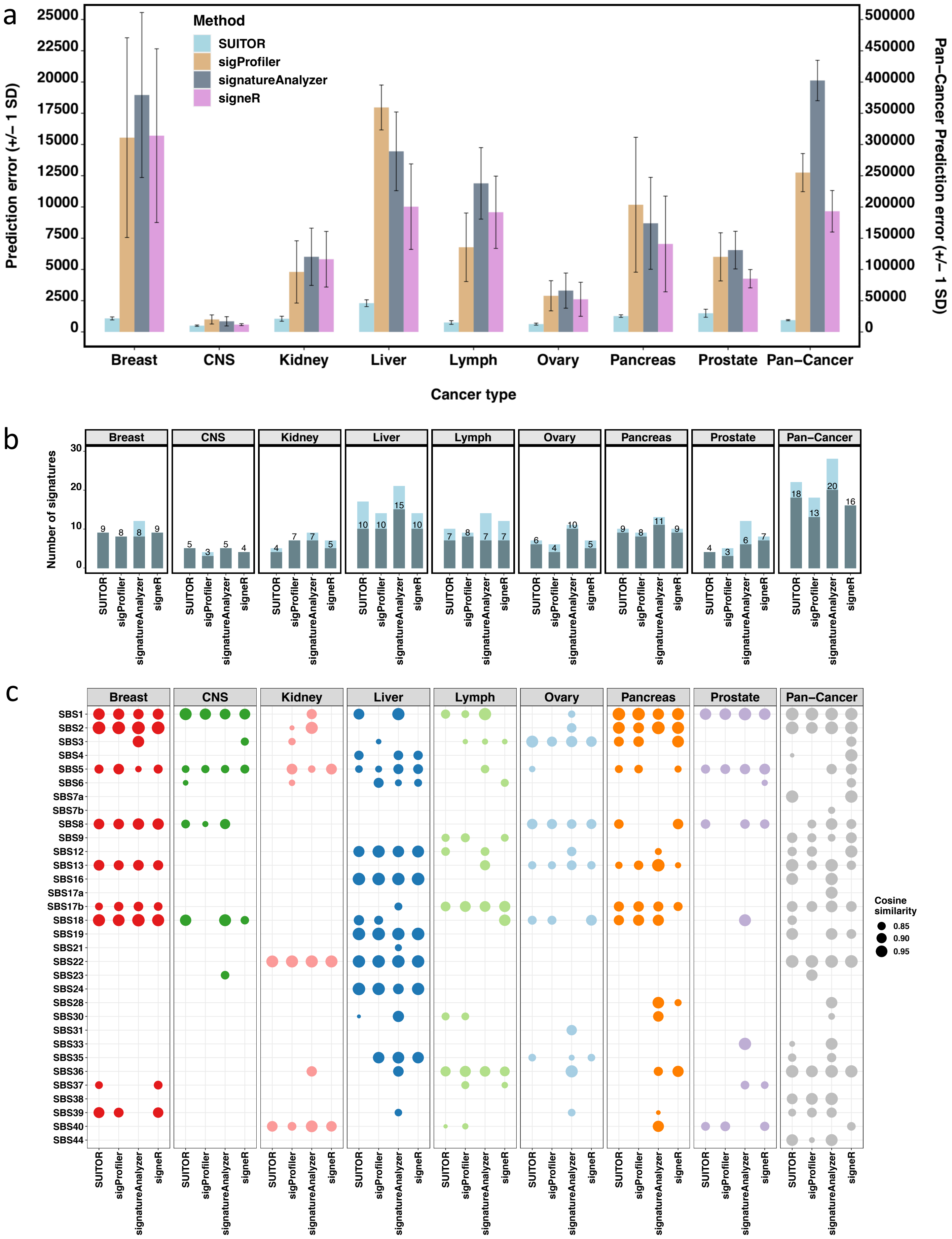

a
Method
SUITOR
sigProfiler
signatureAnalyzer
signeR
Prediction error (+/− 1 SD)
Pan−Cancer Prediction error (+/− 1 SD)
25000
22500
20000
17500
15000
12500
10000
7500
5000
2500
0
500000
450000
400000
350000
300000
250000
200000
150000
100000
50000
0
Breast
CNS
Kidney
Liver
Lymph
Ovary
Pancreas
Prostate
Pan−Cancer
Cancer type
b
Number of signatures
30
20
10
0
c
SBS1
SBS2
SBS3
SBS4
SBS5
SBS6
SBS7a
SBS7b
SBS8
SBS9
SBS12
SBS13
SBS16
SBS17a
SBS17b
SBS18
SBS19
SBS21
SBS22
SBS23
SBS24
SBS28
SBS30
SBS31
SBS33
SBS35
SBS36
SBS37
SBS38
SBS39
SBS40
SBS44
Cosine similarity
0.85
0.90
0.95

Figure 5

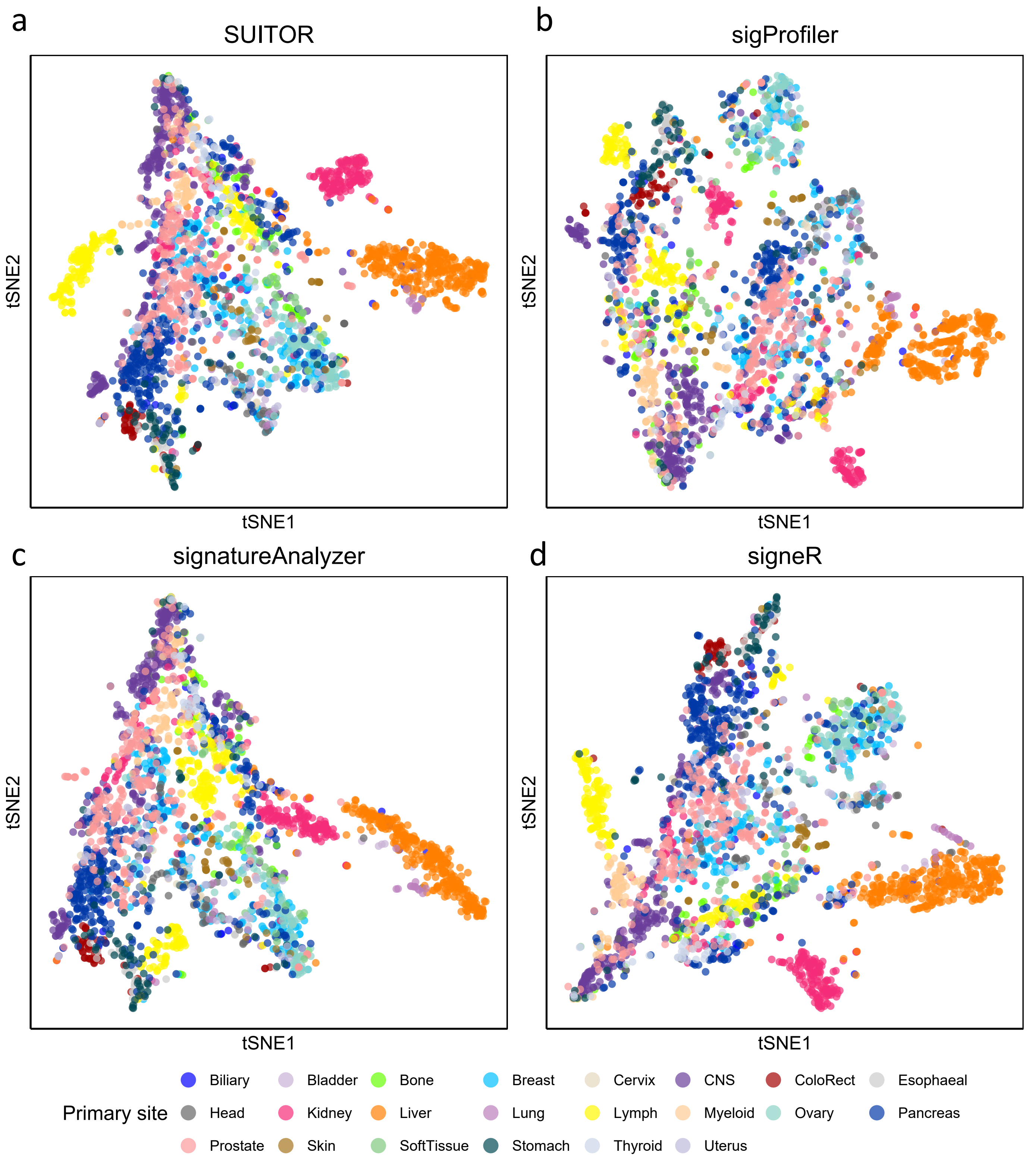

a
SUITOR
b
sigProfiler
c
signatureAnalyzer
d
signeR
tSNE1
tSNE2
Primary site
Biliary
Bladder
Bone
Breast
Cervix
CNS
ColoRect
Esophaeal
Head
Kidney
Liver
Lung
Lymph
Myeloid
Ovary
Pancreas
Prostate
Skin
SoftTissue
Stomach
Thyroid
Uterus

Figure 6

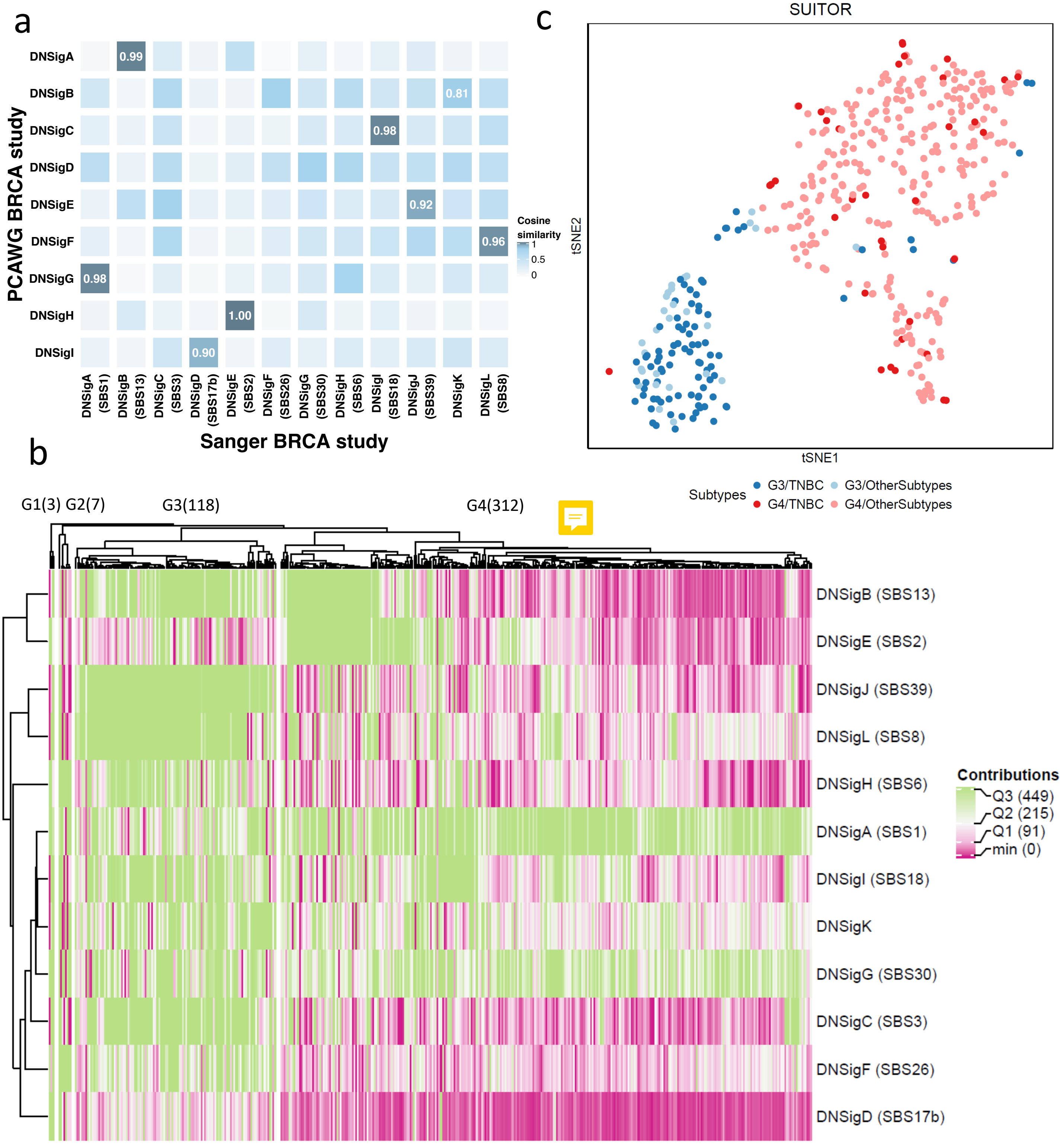
a
PCAWG BRCA study
DNSigA
DNSigB
DNSigC
DNSigD
DNSigE
DNSigF
DNSigG
DNSigH
DNSigI
0.99
0.81
0.98
0.92
0.96
0.98
1.00
0.90
DNSigA (SBS1)
DNSigB (SBS13)
DNSigC (SBS3)
DNSigD (SBS17b)
DNSigE (SBS2)
DNSigF (SBS26)
DNSigG (SBS30)
DNSigH (SBS6)
DNSigI (SBS18)
DNSigJ (SBS39)
DNSigK
DNSigL (SBS8)
Sanger BRCA study
Cosine similarity
1
0.5
0
c
SUITOR
tSNE2
tSNE1
Subtypes
G3/TNBC
G3/OtherSubtypes
G4/TNBC
G4/OtherSubtypes
b
G1(3) G2(7)
G3(118)
G4(312)
DNSigB (SBS13)
DNSigE (SBS2)
DNSigJ (SBS39)
DNSigL (SBS8)
DNSigH (SBS6)
DNSigA (SBS1)
DNSigI (SBS18)
DNSigK
DNSigG (SBS30)
DNSigC (SBS3)
DNSigF (SBS26)
DNSigD (SBS17b)
Contributions
Q3 (449)
Q2 (215)
Q1 (91)
min (0)

Supp figures

Click here to access/download
Supporting Information
Supplementary figures.pdf

Supp tables

Click here to access/download
Supporting Information
Supplementary tables.xlsx

Supp notes

Click here to access/download
Supporting Information
Supplementary notes.pdf
