# Supplementary material for: SUITOR: Selecting the number of mutational signatures through cross-validation
Source: PLoS Comput Biol. 2022 Apr 4;18(4):e1009309. doi: 10.1371/journal.pcbi.1009309 (PMC9009674; doi:10.1371/journal.pcbi.1009309)
Supplement: Attachment — Submitted filename: ResponseLetter_v2.pdf [file pcbi.1009309.s018.pdf]

Response to Referee #1:
We appreciate your insightful and constructive comments. We have revised the manuscript accordingly and provide point-by-point responses below.

1. *It would be best to separate the highlighted area and re-structure it as an independent sentence. (page 9)*
   Response: We followed this comment and re-structured it as an independent sentence. (lines 196-199 on page 9 of the clean version)

2. *This sentence should be re-structured to clarify that liver tumors were separated from the other tumors across all of the t-SNE plots that were generated from the different signature prediction methods. (page 12)*
   Response: This sentence was re-structured as suggested. (lines 322-323 on page 15 of the clean version)

3. *The font size should be altered to remain consistent with the remainder of the text. (page 12)*
   Response: The font size was altered. (line 336 on page 16 of the clean version)

4. *Fig. 6a does not make it apparent that four additional signatures were found by SUITOR in the Sanger BRCA study. Please either remove this or create a separate figure that demonstrates this. (page 13)*
   Response: It's a good point. We revised Fig 6a and the four additional signatures detected in the Sanger BRCA study were currently annotated by the red rectangular frames around the names of signatures. (lines 905-907 on page 37 of the clean version)

5. *It may be best to replace this semicolon with a period and leave the following text as a separate sentence. (page 13)*
   Response: A period is used. (line 349 on page 16 of the clean version)

6. *It may be best to replace "few" with "a few" in the highlighted text. (page 13)*
   Response: We replaced "few" with "a few". (line 350 on page 16 of the clean version)

7. *For reader comprehension, it would be best to alter this text to "eight in vivo" rather than "in vivo eight". (page 14)*
   Response: It was altered. (line 376 on page 17 of the clean version)

8. *In this instance, "hold" should likely be replaced with "held". (page 16)*
   Response: It was replaced. (line 463 on page 21 of the clean version)

9. *This hyperlink leads to a biography that contains a further hyperlink to the data. This additional hyperlink did not effectively re-direct the web-page to the data. Please ensure that a functional hyperlink, which leads directly to the data, is provided. (page 23)*
   Response: We replaced with a hyperlink which leads directly to the dataset. (lines 661-662 on page 30 of the clean version)

10. *Within the highlighted area, "paratheses" should be corrected to "parentheses". (page 28)*

Response: It was corrected. (line 872 on page 36 of the clean version)

11. *In the description of Supplementary Table 3, "signarues" should be corrected to "signatures".*
    Response: This supplementary table is deleted since the random signature simulation was removed, following comments from reviewer #2 and #3.

12. *In Supplementary Table 4, row "SBS40", column "nonzeros SD", please ensure that this NA value is correct.*
    Response: SBS40 contributes one tumor and therefore standard deviation cannot be computed. We added this explanation to Supplementary Table 4. (Now it is Supplementary Table 3.)

13. *For supplementary tables 5, 6, 7, 8, 10, 11 and 12, please ensure that the significance of highlighted values is explicitly stated in the table caption.*
    Response: We revised the table caption and stated explicitly the meaning of highlighted values. (Now they are Supplementary Tables 7, 8, 9, 10, 12, 13 and 14.)

14. *For supplementary tables 5, 6, 7, 8, 9, 10, 11 and 12, please ensure that the significance of color coding for highlighted values (red versus blue) is explicitly stated in the table caption.*
    Response: Following this comment, we explained the color coding in the table caption. (Now they are Supplementary Tables 7, 8, 9, 10, 12, 13 and 14.)

15. *For supplementary tables 5, 6, 8, 9, 10, 11 and 12, please ensure that the highlighted values are consistently displayed in either a bold typeface or non-bold typeface.*
    Response: All highlighted values were displayed in the non-bold typefaces now.

16. The distinction of signatures in parentheses versus signatures not in parenthesis should be explained here. (Supp. Fig. 8)
    Response: We explained the distinction to the Supp. Fig. 8 (currently ordered as Supp. Fig. 10). "The signatures of the Sanger BRCA study are annotated by COSMIC signatures (if cosine similarities > 0.8) among parentheses. For example, DNSigA (SBS1) refers to de novo signature A (DNSigA) being annotated by COSMIC signature SBS1."

Response to Referee #2:
We appreciate your insightful and constructive comments. We have revised the manuscript accordingly and provide point-by-point responses below.

Major comments
In my opinion, the authors did a great job in addressing one of the key points in this relatively new type of genomic analysis, since many of the previously published tools have failed in solving it. Actually, there are a substantial number of R-based tools for mutational signatures analysis recently published, including MutationalPatterns (doi: 10.1186/s13073-018-0539-0), MutSpec (doi: 10.1186/s12859-016-1011-z), SomaticSignatures (doi: 10.1093/bioinformatics/btv408) or SignatureToolsLib (doi: 10.1038/s43018-020-0027-5), that do not provide automatic selection of the number of signatures. Therefore, these tools leave one of the key parameters of the analysis directly on the users' hands. However, many times users are not experts on the computational engines behind the signature extraction process. In this regard, SUITOR appears as a very interesting option to work alongside these other tools, in order to correctly select the number of signatures that better represent the data.
Response: We greatly appreciate that the reviewer recognized our contributions.

However, the authors have not explored this possibility in the manuscript, focusing on benchmarking their tool against three other tools that do include automatic selection of the number of signatures. Although the approach for the selection of the number of signatures provides a new perspective with the insightful use of cross-validation, the tool suffers a lack of completeness in comparison to the other three tools benchmarked in the manuscript, SigProfilerExtractor, SignatureAnalyzer and SigneR. SUITOR does not include almost any user guide, explanation of the input parameters and output files/variables generated, plotting capabilities, possibility of extracting signature and activity matrices, comparison to existing reference signatures (e.g., COSMIC database) or algorithm tuning. Even though the reported benchmarking results are promising, the authors should consider including some of these features to facilitate the usage of SUITOR. Currently, I was not able to perform a simple analysis with the provided test data and obtaining the extracted mutational signatures. I correctly obtained the suggested number of signatures, but not the actual signature profiles.
Response: Thanks for providing insightful suggestions to improving the tool. Following these suggestions, we have made a major update of github SUITOR website: https://github.com/binzhulab/SUITOR and provided a user guide https://github.com/binzhulab/SUITOR/blob/master/User_Guide_SUITOR.pdf. They explained the input parameters and output files/variables of the function to select the number of signatures and how to change the default parameters. In addition, they demonstrated how to extract signature and activity matrices, compare to COSMIC signature database and how to make signature profile plots.

1. *The main concern regarding the presented bioinformatic tool, SUITOR, is that is unclear if it can be used for a complete mutational signature extraction analysis or only for the selection of the number of signatures. According to the provided benchmarking figures and tables, as well as the description of the ECM algorithm, it seems that SUITOR can extract mutational signatures by itself. However, the R code following the instructions provided does not allow to generate the matrices of signatures and activities, giving only the suggested number of signatures as result.*

Response: The reviewer is correct that SUITOR could extract mutational signature profiles and signature activities, which were not emphasized in the previous version of user instructions. Following this comment, we have made a major revision of the user instructions and added in more features, including extracting signatures, estimating activity matrices, and making signature profile plots, among others. See more details at the Github SUITOR page (https://github.com/binzhulab/SUITOR) and the user guide (https://github.com/binzhulab/SUITOR/blob/master/User_Guide_SUITOR.pdf).

2. *The output matrices for the benchmarking are not available (only the cosine similarities in the supplementary tables), which prevents reproducing the reported extraction results for SUITOR or the other three tools.*
Response: We have submitted the output matrices of the benchmarking as a supplementary dataset.

3. *The rationale for the selection of the benchmarking datasets is not provided in some cases, e.g., selection of SBS8 for the in silico simulations with one signature, or exclusion of hypermutated tumors with >10 mutations/Mb in PCAWG and breast cancer data analysis. Actually, hypermutated tumors are particularly important in mutational signature analysis since they can lead to the extraction of false positive signatures (doi: 10.1038/s41586-020-1943-3). Therefore, their influence on SUITOR performance should be assessed.*
Response: We added the rationale of selection of SBS8 for *in silico* simulations in Methods section. Specifically, SBS8 signature is dominated by C>A and T>A, but not as flat as SBS3 or SBS5 and not as spiky as SBS1 or SBS2. Hence, it's not too hard or too easy to be detected and was selected as the simulation signature. (lines 584-585 on page 26 of the clean version)

For analyzing PCAWG study and Sanger breast cancer study, we excluded hypermutated tumors with mutation burden more than 10 mutations/Mb. Most of caner types we analyzed have just few hypermutated tumors (e.g., we excluded 5 tumors of hepatocellular carcinoma, 4 tumors of pancreatic adenocarcinoma, 4 tumors of breast adenocarcinoma, 1 tumor of renal cell carcinoma,1 tumor of ovary adenocarcinoma, 7 tumors of B-cell non-Hodgkin lymphoma and zero tumors for other two cancer types). Hence, inclusion of hypermutated tumors or not would not likely impact the extracted signatures for these eight cancer types.

Moreover, we further analyzed a couple of cancer types with more prevalent hypermutated tumors. SUITOR would find less signatures when we included hypermutated tumors (Supplementary table. 17). For example, only SBS6 (associated with defective DNA mismatch repair) and SBS10a (associated with polymerase epsilon exonuclease domain mutations) could be detected for colorectal adenocarcinoma when including hypermutated tumors (17 over 60 tumors). It suggests the inclusion of hypermutated tumors would likely hinder the detection of other signatures by SUITOR, as least when the number of tumors is small. (lines 405-415 on page 19 of the clean version)

4. In addition, the authors should make a clear distinction between benchmarking, which is only possible in silico using simulated data, and exploratory analysis using real data (either in vitro or in vivo). Benchmarking is only possible in simulations because ground truth signatures are needed for comparison, and these are not available in real data.

Response: Following this suggestion, we made a clear distinction between benchmarking. In the beginning of the "*in silico* simulation studies" section, we emphasized that "To benchmark the performance of SUITOR and other methods, we evaluated them through additional *in silico* simulations, for which the estimated signatures are compared to the given signatures as the ground truth." (lines 231-233 on page 11 of the clean version)

5. Also, ground truth signature/s are not available for the first in silico analysis performed in the manuscript (lines 436-440) since the input matrix was constructed using random numbers, instead of generating a random ground truth signature/s. Moreover, the authors claimed that the signature extracted from this random-generated input matrix is similar to SBS3 (line 215), which is probably related to the fact that SBS3 is a flat-like signature. However, SBS3 has been previously associated with defective homologous recombination-based DNA damage repair and germline mutations in BRCA1 and BRCA2 (doi: 10.1016/j.cell.2012.04.024, 10.1038/nature17676, 10.1038/nm.4292, 10.1038/onc.2016.243 and others).
Response: Following this suggestion, we removed this simulation and added another more comprehensive and realistic one. In the main text, a new paragraph was added as follows.

"Finally, we investigated the setting when part of somatic mutations was called by mistake, which likely occurs in practice due to sequencing and/or calling errors. We simulated a mutation type matrix for 300 tumors of eight signatures which cover six major substitution types. In addition, we added mutation counts caused by errors for each tumor generated from a uniform distribution with various error levels (details in Methods). We repeated this simulation by 20 times. Notably, SUITOR consistently detect eight signatures regardless of error levels (Fig. 3b); in contrast, although SBS9 was detected by SigProfilerExtractor (5 over 20 replicates), SignatureAnalyzer (20 over 20 replicates), and signeR (1 over 20 replicates) when there are no mutation calling errors, it was rarely detected when mutation calling errors present; for example, when the error level is 1.2, all three methods could not detect SBS9. Besides SBS9, signature SBS39 was missed by signeR when there existed error mutations. Compared to the given signature profiles, cosine similarities of signatures detected by SUITOR were higher than 90% for SBS4, 6, 7a, 17b, 22, 26 and 39 and slightly lower than 90% for SBS9 (Supplementary Fig. 9b). For this simulation involved mutations called by errors, we further evaluated the performance of CV2K and SparseSignatures. When there were no mutation calling errors, CV2K was able to detect true 8 signatures among 19 of 20 replicates; however, its performance declined with increasing error levels; when the error level is at 1.2, CV2K detected 8 signatures in 6 of 20 replicates and detected 7 signatures in other 14 replicates (Supplementary table. 4). SparseSignatures tended to find more signatures even without mutation calling errors; it detected more than 12 signatures (as many as 20 signatures for one replicate) in 9 of 20 replicates; when the error level was 1.2, SparseSignatures identified the true 8 signatures in only 2 replicates (Supplementary table. 4)." (lines 261-282 on pages 12-13 of the clean version)

The details of simulation were provided in Methods section as follows.

"*In silico* simulation of eight signatures with some mutations called by mistake
We simulated a mutation type matrix for 300 tumors with mutation calling errors. We repeated this simulation 20 times and analyzed the simulated type matrices by SUITOR, SigProfilerExtractor, SignatureAnalyzer, signeR, CV2k and sparseSignatures. We chose eight

signature profiles as the true signature profiles for **W** to cover all six major substitution types: SBS4 for [C > A], SBS39 for [C > G], SBS6 and SBS7a for [C > T], SBS22 for [T > A], SBS26 for [T > C], SBS17b for [T > G] and SBS9 for [T > C] and [T > G]. The activity matrix **H** was first generated from a uniform distribution with the range [0, 100], then some randomly chosen elements in the activity matrix **H** were set to be zero to mimic the real data. The true mutation type $\mathbf{V^0}$ without error was generated by a Poisson distribution with mean **WH**. In addition, we added error mutation counts for each mutation type and each tumor; error mutation counts were generated from a uniform distribution with the range [0, **a**×**b**], where the error level **a** equates to 0 (i.e., no error mutation counts), 0.4, 0.8 and 1.2, and **b** is the average mutation count of each tumor (i.e., **b** being the column average of the matrix $\mathbf{V^0}$).” (lines 609-622 on pages 27-28 of the clean version)

6. *The authors should clarify why the PCAWG benchmarking on the three additional tools was performed on 90% of the counts in the mutation catalog matrix (lines 482-491) and not on 100% as it was done in the in vitro benchmarking (lines 410-412). This cross-validation approach may have biased the benchmarking results since the other tools are not designed for dealing with missing data. The authors used the medians of available mutation counts per mutational context to fill the missing data, which may have disrupted the original input matrix and the whole extraction process.*
Response: Thanks for pointing this out. In fact, 90% of the counts in the mutation catalog matrix was used for comparing the prediction errors only, for which 10% of the counts musted be hold out for each method to avoid overfitting. For other comparisons, such as comparing the methods’ ability to infer the number of signatures and their profiles (Fig. 4b and 4c), 100% of the counts in the mutation type matrix were used for SigProfilerExtractor, SignatureAnalyzer and signeR to make a fair comparison. The reviewer is right that the other tools are not designed for dealing with missing data and hence cannot do cross-validation, which motivated us to develop SUITOR. We added this clarification to both the main text and method section. (lines 299-303 on page 14 of the clean version; lines 653-657 on pages 29-30 of the clean version)

7. *As previously mentioned, the GitHub README file is missing different sections, in particular a detailed explanation of the different input parameters, as well as the output variables and objects. A more detailed vignette would also be useful apart from the html file provided (https://github.com/binzhulab/SUITOR/blob/master/SUITOR.html), in a similar way as the SigneR authors presented for their tool (https://www.bioconductor.org/packages/release/bioc/vignettes/signeR/inst/doc/signeR-vignette.html). An example of a comprehensive mutational signature extraction analysis should be included in this vignette in order to be reproducible by potential users.*
Response: We have made a major overhaul of SUITOR github website. The current website (https://github.com/binzhulab/SUITOR) provides a detailed explanation of the installation of the R package and various input parameters. The user guild (https://github.com/binzhulab/SUITOR/blob/master/User_Guide_SUITOR.pdf) further explains the output variables and offers a compressive analysis example, including selection of the number of signatures, extraction of signature profiles and activities, creation of the prediction error plot, comparison to the COSMIC signature dataset and generation of signature profile plots.

8. *In a SUITOR test following the code provided in the html file and using the provided test data but fixing the print parameter on print=3, it is concerning to observe that some iterations of the EM algorithm did not converge. It is not clear in the manuscript how many iterations are used as maximum for the multiplicative updates/ECM algorithm, as well as how many NMF/ECM replicates are considered.*
Response: All the results described in the manuscript are based on the 2000 maximal iterations. If the maximal iteration was reached, the function would produce a warning message "The maximal iteration has been reached and the algorithm may not converge yet; please increase the R function parameter: maximal iteration *max.iter*." and we would increase the maximal iteration. In the updated user guild, the default maximal iteration is set at 2000 and users could change it as a R function parameter. (line 523 on page 24 of the clean version)

Minor comments:

1. *The authors should clarify why 0.8 was used as the threshold for cosine similarity matching instead of the 0.9 commonly used in the prior literature (doi: 10.1038/s41586-020-1943-3).*
Response: Following this comment, we clarified the choice of 0.8 as the threshold for cosine similarity in Methods section. "The signatures with cosine similarity larger than 0.8 were reported. The higher the cosine similarity the better match to a COSMIC signature profile. The cosine similarity equivalent to one denotes a perfect match. Note that the cosine similarity larger than 0.9 is a more stringent cutoff and our simulation studies show few true signatures may be missed by using the cosine similarity 0.9 as the cutoff." (lines 638-642 on page 29 of the clean version)

2. *The inclusion of the developed package in one of the main repositories for R packages (R CRAN or Bioconductor) is recommended since this has also been done by many of the other R-based mutational signature analysis tools already available in the field, including MutationalPatterns, SomaticSignatures or SigneR.*
Response: We have submitted the SUITOR R package to Bioconductor and are waiting the approval from Bioconductor.

3. *The background section is missing the newest collection of reference mutational signatures in the COSMIC database, corresponding to version 3.2 from March 2021 (https://cancer.sanger.ac.uk/signatures/). The authors should also mention in the introduction the other mutational types where mutational signatures have been extracted, including indels, doublet base substitutions (reference signatures also present in COSMIC), structural variants (doi: 10.1038/nature17676 among others) and copy number variants (doi: 10.1038/s41588-018-0179-8, 10.1101/2021.04.30.441940 and others).*
Response: These are good points. We added the references of COSMIC database version 3.2, indels, doublet base substitutions, structural variants and copy number variants in Background section. (lines 53-56 on page 3 of the clean version)

4. *The association of NTHL1 germline mutations with a particular mutational signature (SBS30) in more than 10 different cancer types should also be included in the background section in line 34 apart from the association with breast cancer (doi: 10.1016/j.ccell.2018.12.011).*
Response: The new reference has been cited in Background section. (line 58 on page 3 of the clean version)

5. *The claim about the method used by SomaticSignatures to select the number of signatures (lines 44-46) is inaccurate, since this tool does not have an automatic method for this selection. The authors correctly pointed out that the authors of SomaticSignatures suggested using the residual sum of squares and the explained variance. However, this is only a suggestion, and the user is required to manually choose the number of signatures to use in the extraction based on a set of plots depicting the mentioned metrics as well as some additional ones.*
Response: We followed this comment and updated the summery sentence of SomaticSignatures. (lines 72-74 on pages 3-4 of the clean version)

6. *The language used for the first explanation of the specific cross-validation scheme developed for the mutational signature analysis problem in lines 77-83 is unclear, making it difficult to follow.*
Response: This is a valid point. We removed the technical details and instead emphasized that the standard cross-validation scheme for supervised learning is inapplicable for the unsupervised mutational signature analysis. (lines 103-110 on page 5 of the clean version)

7. *In the mathematical notation of the method, W and H matrices are switched with regard to the original implementation of the mutational signatures framework (doi: 10.1016/j.celrep.2012.12.008 and 10.1038/s41586-020-1943-3). W was originally defined as the matrix of signatures (rows = number of mutational contexts & columns = signatures) and H as the matrix of activities/exposures (rows = signatures & columns = samples). In the manuscript, the authors changed the notation and transposed the matrices, which is mathematically correct but not in agreement with previous literature.*
Response: Following this comment, we switched the mathematical notation of the matrices **W** and **H** to agree with the previous literature across the manuscript.

8. *The authors should clarify which data was used for generating the clustering presented in figure 5 and in the results section (lines 254-256).*
Response: The clustering is based on signature activities **H** estimated by each method. We clarified it in the manuscript. (lines 317 and 321 on page 15 of the clean version)

9. *The authors should provide some evidence that SUITOR is able to handle the selection of the number of signatures arising from other genomic alterations (as they claimed in the discussion section, lines 312-314) since only data from single base substitutions (SBS96 context) is presented in the manuscript.*
Response: Following this comment, we applied SUITOR for the selection number of INDEL

signatures. SUITOR can detect four INDEL signatures in ovarian adenocarcinoma. Three of them are similar to COSMIC INDEL signatures: ID1 (associated with replication slippage, cosine similarity = 0.97), ID6 (associated with defective homologous recombination-based DNA damage repair, cosine similarity=0.95), ID8 (associate with DNA double-strand breaks repair by non-homologous end joining, cosine similarity=0.88). We added these results to the manuscript. (lines 384-401 on page 18 of the clean version)

10. *The parameters used for the different tools are missing in the methods section, in particular, the number of NMF/ECM runs performed and the maximum number of iterations per run in case that convergence is not reached, including the ones used for SUITOR (lines 393-397).*
Response: We added parameters used for the different tools in Methods section: "Parameters used for SUITOR and other mutational signatures analysis tools". (lines 518-535 on page 24 of the clean version)

11. *SigProfilerExtractor should be used as the name of the corresponding tool instead of sigProfiler, according to the information present in the GitHub provided in line 510 and also by the authors in their preprint for that particular tool (doi: 10.1101/2020.12.13.422570).*
Response: We followed this comment and used the name "SigProfilerExtractor" in the manuscript across the manuscript.

12. *Since the specific version number is not available, an approximate date of download should be included for SignatureAnalyzer in line 512.*
Response: We added an approximate download date. (line 679 on page 31 of the clean version)

13. *The authors should consider moving some technical details of the results-overview section (lines 97-156) to the methods section.*
Response: We moved technical details to Methods section. (lines 429-445 on pages 19-20 of the clean version)

14. *A citation to the COSMIC database (e.g. doi: 10.1093/nar/gky1015) should be included in the first mention of the COSMIC reference mutational signatures, as well as in line 32 when mentioning the current reference set of mutational signatures. The URL https://cancer.sanger.ac.uk/signatures/ may also be included.*
Response: The citation is now included in both places and the URL is added to section of "Software and dataset webpages". (line 56 on page 3 of the clean version)

15. *A citation to a review about the several mutational signature analysis tools in the literature should be added in lines 42-43. Some examples are doi: 10.1093/bib/bbx082, 10.1016/j.mam.2019.05.002 or 10.1371/journal.pone.0221235. In addition, the authors should*

mention the two main types of analysis that have been addressed by prior studies: signature extraction and signature refitting based on reference mutational signatures. Whereas the authors have focused their manuscript on the extraction process, it is important to mention the availability of the refitting analysis, with potential applicability in clinical practice (as discussed here, doi: 10.1186/s13059-016-0893-4).
Response: We appreciated these comments and mentioned that there are two types of mutational signature analysis and have cited the recommended review references. The availability of refitting analysis method is emphasized and the reference of deconstructSigs was cited. We revised the second paragraph on the page two as follows.

"There are two main types of mutational signatures analysis[21-23]: signature extraction and signature refitting. Signature extraction aims to extract *de novo* signature profiles[24-28] while signature refitting to estimate signature activities based on reference mutational signatures with potential applicability in the clinical setting[29,30]. Our interest is on selecting the correct number of *de novo* mutational signatures for signature extraction in cancer genomics studies[31,32], which has been insufficiently explored." (lines 67-72 on page 3 of the clean version)

Response to Referee #3:
We appreciate your insightful and constructive comments. We have revised the manuscript accordingly and provide point-by-point responses below.

1.This paper addresses a key challenge in mutational signatures analysis of cancer genomes that is determining the correct number of distinct mutational processes/signatures operating on studied samples, i.e. the rank of factorization. The problem is important, especially that it significantly affects the downstream analyses. The method is based on the cross-validation method used for model selection. The authors evaluate their method on simulated and in vitro data, as well as real cancer samples. They compare it with existing methods developed for signature inference (not specifically for the identification of number of signatures).
Response: We greatly appreciate that the reviewer recognized our contributions and provided insightful suggestions to further improve the proposed method.

2. Overall the manuscript is well written and presents an interesting approach, but it lacks clarity in some parts. The significance of the study lies in stating an important practical problem directly and proposing a method to solve it. However, it seems that the authors are not aware of existing approaches for this problem that use cross-validation as well. CV2K method (Gilad et al, Mach. Learn. Sci. Technol., 2020) uses a similar cross-validation schema (although random, not balanced separation as the authors) combined with sBCD NMF. SparseSignatures (Lal et al., PLoS Comp. Biol. 2021; available in BioRxiv and Bioconductor since 2018), a framework to extract signatures, uses cross-validation to identify the number of signatures. These two methods seem to be more appropriate for benchmarking the method, as they use a similar cross-validation idea (both methods) or were developed specifically for the same problem (CV2K).
Response: We appreciate the reviewer's suggestion of these two methods.

First, we cited them in the introduction. "Recently, other cross-validation methods have been proposed to select the number of signatures; compared to SUITOR, CV2K focuses on selecting the number of signatures only (without extracting signature profiles and estimating signature activities) and is based on random, not balanced separation[41]; SparseSignatures applies cross-validation to select both the number of signatures and the shrinkage parameter simultaneously, which is computationally intensive and tends to infer signatures with spiking profiles[42]." (lines 120-126 on page 6 of the clean version)

Second, CV2K and SparseSignatures were compared with SUITOR in the *in silico* simulations "For this simulation involved mutations called by errors, we further evaluated the performance of CV2K and SparseSignatures. When there were no mutation calling errors, CV2K was able to detect true 8 signatures among 19 of 20 replicates; however, its performance declined with increasing error levels; when the error level is at 1.2, CV2K detected 8 signatures in 6 of 20 replicates and detected 7 signatures in other 14 replicates (Supplementary table. 4). SparseSignatures tended to find more signatures even without mutation calling errors; it detected more than 12 signatures (as many as 20 signatures for one replicate) in 9 of 20 replicates; when the error level was 1.2, SparseSignatures identified the true 8 signatures in only 2 replicates (Supplementary table. 4)." (lines 274-282 on page 13 of the clean version)

Finally, CV2K and SparseSignatures were evaluated and compared for the PCAWG studies. "In addition, we applied CV2K and SparseSignatures to select the number of signatures. The numbers selected by CV2K were comparable to ones by SUITOR (e.g, CV2K: 8 signatures vs SUITOR: 9 signatures for breast cancer; CV2K: 4 signatures vs SUITOR: 4 signatures for prostate cancer; Supplementary table. 5). Since CV2K does not infer signatures profiles, we were unable to evaluate their similarities to COSMIC signatures. SparseSignatures tended to detect slightly fewer signatures than other methods (Supplementary table. 5); since SparseSignatures imposed the sparsity assumption, its inferred profiles were spiky and less similar to the COSMIC signatures (Supplementary table. 6)." (lines 326-333 on page 14 of the clean version)

3. Please do not change existing terminology used in signature analysis literature; see the seminal paper by Alexandrov et al. (Cell Reports, 2013) for reference. It can lead to misunderstanding and confusion. For example, a mutational catalog of a cancer genome is defined by Alexandrov et al. as a vector containing the number of somatic mutations of a genome defined over a finite alphabet of mutation types. Thus, "A[C>G]G" is a mutation type not a mutation catalog; and there are 96 mutation types (or categories, features) not catalogs.
Response: We followed this comment and used the term 96 mutation types, instead of catalogs. Other changes were made to be consistent with the notations used in Alexandrov et al. (Cell Reports, 2013). For example, we now used the column of mutation type matrix to represent a subject across the manuscript.

4. The authors use two in vitro studies to benchmark their approach. In both of these studies there is only one perturbation per experiment (gene knockout or cell exogenous exposure) leading to at most one mutational signature beyond the background. However, there is an experimental dataset available that has both gene knockouts and genotoxins, see the paper by Volkova et al. (Nature Communications, 2020). The authors could evaluate their approach on this dataset if possible (e.g. samples that have high number of mutations).
Response: We are very interested in testing this dataset. However, as reviewer may notice, this dataset is based on C. elegans, whose genome size is around 101 MB. In addition, its mutation rate is quite low with median mutation burden 0.1 mutations/Mb. Consequently, the mutation counts are very low relative to the counts of somatic mutations of human tumors based on WGS (commonly thousands of mutations). The SUITOR and other methods developed for human tumors seem inapplicable to the raw dataset directly. Nevertheless, we combined 100 C. elegans together to increase the mutation counts as a group (leading to 27 groups for 2717 C. elegans). We added new results to Discussion section as follows.

"We applied SUITOR to an experimental study on C. elegans[47]. Since mutation counts of C. elegans are very low (partially due to its relative smaller genome size around 101 Mb) relative to the mutation counts of human tumors based on WGS (human genome size around 3,000 Mb), we combined 100 C. elegans together to increase the mutation counts as a group (leading to 27 groups for 2717 C. elegans). SUITOR could detect five *de novo* SBS signatures, including one similar to SBS7c (associated with UV-light as a genotoxic exposure, cosine similarity=0.90) and another one similar to SBS30 (associated with deficiency in base excision repair due to gene knockout in this experimental study, cosine similarity=0.86)." (lines 391- 401 on page 18 of the clean version)

5. Is there a particular reason that the two in vitro datasets were treated in a different way? The samples from Kucab et al. were all analysed together (put in a single V matrix), while the samples from Zou et al were split into two analyses (MSH6 knockout and the rest). All methods should be able to find two signatures in Zou et al even when all samples were analysed together. Only MSH6 KO samples have two signatures present while all other samples should have only one signature (background) with non-zero activity.

Response: Our intension was to begin with a relatively simple *in vitro* study to evaluate the true positive and true negative separately. We first evaluated the in vitro CRISPR-Cas9-mediated knockout study of the DNA repair gene MSH6, which was known to induce a detectable signature and hence could be used for evaluation as the positive controls; Next, we extracted signatures of knockout studies of six DNA repair genes (CHEK2, NEIL1, NUDT1, POLB, POLE and POLM), which did not induce experimentally detectable signatures and hence could be used for evaluation as the negative controls. We have clarified this point in the manuscript. (lines 180-192 on pages 7-8 of the clean version)

6.For the second in vivo dataset (Kucab et al), all methods found a different number of signatures. However, how many signatures should be expected for this dataset? Have you tried to see how many profiles of these 28 mutagens are similar to each other? If you assume 0.8 cosine similarity (as you do for signature comparison), how many clusters of mutagen profiles would you have?

Response: The stable mutational signatures were experimentally identified for 28 mutagens. Note that the signature profiles characterized by experiments are not all distinct. Hierarchical clustering of 28 signature profiles suggests some signature profiles are clustered together (Supplementary Fig. 11a) with 13 clusters based on cosine similarity >0.8 between profiles; consensus clustering indicates that 13 clusters of signature profiles demonstrated a stable clustering (consensus matrix in Supplementary Fig. 11b). We added these new results to Methods section. (lines 567-572 on pages 26 of the clean version)

7. Moreover, have you inspected the exposure matrix inferred by different methods? Each studied sample comes from a single mutagen treatment, so its profile should be affected by at most two signatures (background and mutagen signature). Thus, the exposure matrix (for all samples as you have in your paper) should be very sparse with at most two non-zero values in each row; more non-zero values would suggest overfitting and the number of signatures is too high and should be reduced. This could lead to a good validation of the result.

Response: This is a good point. We have inspected the mutation activity matrices inferred by different methods and added more details to the revised manuscript. We found that in general the activities of the signature of the exposed mutagen and the background signature were high, while the activities of other signatures of the unexposed mutagen was very low (but not zero exactly), say 0.006; Since there are total 324 tumor subclones, we stratified the tumor subclones based on the signature activities, which was visualized by plots of t-distributed stochastic neighbor embedding (t-SNE).

We updated the results in the manuscript as follows. “Finally, we examined the signature activity matrices which were estimated by each method. Since each tumor subclone was exposed to one exogenous mutagen, we expected that the signature activities of the corresponding mutagen and the background signature should be high, while the activities of other signatures should be close to zero (but not necessarily as exactly zeros); consequently, when we clustered tumor subclones based on signature activities, tumor subclones exposed to the same mutagen would be clustered together and separated from others. We found that SUITOR and SignatureAnalyzer were able to separate most subclones into distinct clusters, each corresponding to a unique mutagen exposure (Supplementary Fig. 5). In contrast, SigProfilerExtractor merged subclones exposed to BPDE and dibenz[a,h]anthracene diol-epoxide (DBADE); signeR mixed subclones exposed to N-ethyl-N-nitrosourea (ENU) and 1,2-DMH; and both SigProfilerExtractor and signeR mixed subclones exposed to 9-Nitrochrysense and aristolochic acid I (AAI).” (lines 208-219 on page 10 of the clean version)

8. Figure 2b, top panel, is incorrect. It shows the MSH6 knockout signature not the background signature. Both top and bottom panels (Fig. 2b) show the same signature, but the top panels should show the background signature that is similar to signatures shown in Supplemental Figure 2b, or signature in Fig 3a (parental clone) in the original paper (Zou et al).
Response: The reviewer is right. We have corrected this error and updated the Figure 2b.

9. The random mutation scenario in in silico simulation study seems artificial as it is not similar to any existing COSMIC signature. The proposed association with SBS3 is weak; SBS3 is usually associated with HR deficiency in signature studies. Instead, you should expand your two other scenarios. More signatures could be tested in your design with one signature, not only SBS8. However, a design with more signatures is more realistic and corresponds to actual problems in cancer studies. Thus, you could expand the last scenario in place of the first two. It will be more informative.
Response: Following this suggestion, we removed the random mutation scenario and added few more comprehensive and realistic simulations as follows.

“Finally, we investigated the setting when part of somatic mutations was called by mistake, which likely occurs in practice due to sequencing and/or calling errors. We simulated a mutation type matrix for 300 tumors of eight signatures which cover six major substitution types. In addition, we added mutation counts caused by errors for each tumor generated from a uniform distribution with various error levels (details in Methods). We repeated this simulation by 20 times. Notably, SUITOR consistently detect eight signatures regardless of error levels (Fig. 3b); in contrast, although SBS9 was detected by SigProfilerExtractor (5 over 20 replicates), SignatureAnalyzer (20 over 20 replicates), and signeR (1 over 20 replicates) when there are no mutation calling errors, it was rarely detected when mutation calling errors present; for example, when the error level is 1.2, all three methods could not detect SBS9. Besides SBS9, signature SBS39 was missed by signeR when there existed error mutations. Compared to the given signature profiles, cosine similarities of signatures detected by SUITOR were higher than 90% for SBS4, 6, 7a, 17b, 22, 26 and 39 and slightly lower than 90% for SBS9 (Supplementary Fig. 9b). For this simulation involved mutations called by errors, we further evaluated the performance of CV2K and SparseSignatures. When there were no mutation calling errors, CV2K

was able to detect true 8 signatures among 19 of 20 replicates; however, its performance declined with increasing error levels; when the error level is at 1.2, CV2K detected 8 signatures in 6 of 20 replicates and detected 7 signatures in other 14 replicates (Supplementary table. 4). SparseSignatures tended to find more signatures even without mutation calling errors; it detected more than 12 signatures (as many as 20 signatures for one replicate) in 9 of 20 replicates; when the error level was 1.2, SparseSignatures identified the true 8 signatures in only 2 replicates (Supplementary table. 4)." (lines 261-282 on pages 12-13 of the clean version)

The details of simulation were provided in Methods section as follows.

"***In silico* simulation of eight signatures with some mutations called by mistake**

We simulated a mutation type matrix for 300 tumors with mutation calling errors. We repeated this simulation 20 times and analyzed the simulated type matrices by SUITOR, SigProfilerExtractor, SignatureAnalyzer, signeR, CV2k and sparseSignatures. We chose eight signature profiles as the true signature profiles for **W** to cover all six major substitution types: SBS4 for [C > A], SBS39 for [C > G], SBS6 and SBS7a for [C > T], SBS22 for [T > A], SBS26 for [T > C], SBS17b for [T > G] and SBS9 for [T > C] and [T > G]. The activity matrix **H** was first generated from a uniform distribution with the range [0, 100], then some randomly chosen elements in the activity matrix **H** were set to be zero to mimic the real data. The true mutation type $\mathbf{V}^{\mathbf{0}}$ without error was generated by a Poisson distribution with mean **WH**. In addition, we added error mutation counts for each mutation type and each tumor; error mutation counts were generated from a uniform distribution with the range [0, $\mathbf{a}\times\mathbf{b}$], where the error level **a** equates to 0 (i.e., no error mutation counts), 0.4, 0.8 and 1.2, and **b** is the average mutation count of each tumor (i.e., **b** being the column average of the matrix $\mathbf{V}^{\mathbf{0}}$)." (lines 609-622 on pages 27-28 of the clean version)

10. In Figure 3b, some methods, including SUITOR, find the same signature more than 20 times in 20 replicates. This suggests overfitting as the same COSMIC signature is found more than once. Can it be related to a case when prediction error for a validation set exhibits a "plateau" near the minimum, i.e. similar prediction error for different number of signatures? In such a case it might be reasonable to select the lowest possible number of signatures not the number that gives the lowest error as it might be statistically impossible to differentiate these two cases.
Response: This is a keen observation. We checked cosine similarities of the detected signatures and found something interesting. Note that we included two flat signatures in this simulation. For SUITOR, there exist one replicate where two detected signatures are most similar to SBS5 (cosine similarity 0.97 and 0.91, respectively). It suggests that two flat signatures were detected in this replicate (i.e., SBS5 and SBS40), which is consistent with the simulation design; indeed, the signature with cosine similarity 0.91 to SBS5 is also similar to SBS40 with cosine similarity 0.83. Similarity, signeR found two flat signatures in two replicates. Both flat signatures are most similar to SBS5 followed by SBS40. In addition, signeR found 2 SBS2 signatures in 3 replicates and 2 SBS13 signatures in 2 replicates, suggesting that signeR would occasionally misidentify APOBEC signatures (SBS2 and SBS13). We add these new findings to the manuscript on page 11. (lines 244-250 on pages 10-11 of the clean version)

11. How are the missing values selected in the initial step of the algorithm? In line 376, it is stated that you use the median of mutation counts in V (training) per each mutation catalog (meaning mutation type), i.e. median of non-missing values from a corresponding column of V. However, in the detailed description of the algorithm (Supplementary note 3), it stated that these are the medians from the corresponding row of V. Please fix this (probably in Supplementary note 3) and potentially other places where it is incorrectly stated.

Response: It should be the median of non-missing values of each mutation type and now are described by the method section and supplementary note correctly. Note that following the 7th minor comment of review #2, we transposed the matrix V to agree with previous literature, for which each row represent a mutation type and each column a tumor across the manuscript.

12. The above mentioned initialization is okay for SUITOR as it tries to improve the estimates of the held-out mutation counts. However, it does not seem appropriate to fill in missing values in datasets provided to other methods (SigProfiles, SignatureAnalyser, signeR) as you do in the in vivo (PCAWG) benchmarking. Missing mutation count data is not a problem in mutational signature analysis, so these methods were not designed to deal with it. SUITOR imputes them because it is part of the cross-validation schema it uses. This leads to unfair comparison between SUITOR and other methods. It would be fair if the authors provide to the other methods the values imputed by SUITOR to fill in the held-out mutation counts. This way the comparison between methods would assess the methods' ability to infer the number of signatures and their profiles, not the other methods' inability to correctly infer the missing values and signature profiles based on incorrect input data. As it is, the imputed values are not even normalized to a sample's overall mutation counts; for each mutation type, all missing values are replaced by the same value (column median). Thus, SUITOR has an advantage when compared with other methods.

Response: This is a good point. In fact, the held-out mutation counts in the mutation type matrix was used for comparing the prediction errors only, for which creation of training, validation and testing datasets separately is essential to avoid overfitting. For comparing the methods' ability to infer the number of signatures and their profiles (Fig. 4b and 4c), 100 % of counts in the mutation type matrix were used for SigProfilerExtractor, SignatureAnalyzer and signeR to make a fair comparison. The reviewer is right that the other tools are not designed for dealing with missing data and hence cannot do cross-validation, which motivated us to develop SUITOR. We added this clarification to both the main text and Methods section. (lines 299-303 on page 14 of the clean version; lines 653-657 on pages 29-30 of the clean version)

Minor comments:

- Use correct names for algorithms (upper/lowercase), i.e. SigProfiler, SignatureAnalyser, signeR, consistently.

Response: We now used the names for algorithms consistently as SigProfilerExtractor (following the suggestion by the referee #2), SignatureAnalyzer and signeR across the manuscript.

- line 52: "elaborated" > "are elaborated"

Response: It's changed. (line 81 on page 4 of the clean version)

- line 125: “been” > “be”
Response: It’s changed. (line 442 on page 20 of the clean version)

- line 138: “The” > “the”
Response: It’s changed. (line 149 on page 7 of the clean version)

- line 359: fix the reference
Response: It’s fixed. (line 477 on page 22 of the clean version)
