# Supplementary material for: SUITOR: Selecting the number of mutational signatures through cross-validation
Source: PLoS Comput Biol. 2022 Apr 4;18(4):e1009309. doi: 10.1371/journal.pcbi.1009309 (PMC9009674; doi:10.1371/journal.pcbi.1009309)
Supplement: Attachment — Submitted filename: Response to Referee_Revision2.docx [file pcbi.1009309.s019.docx]

**Response to Referee #2:**
We appreciate your further comments. We have revised the manuscript accordingly and provide point-by-point responses below.

1. As I previously mentioned, the commonly used cosine similarity threshold for mutational signature analysis in previous literature is 0.9. The authors claimed that their simulation studies show minor differences between this well-established threshold and their selected one (0.8). However, they should provide some detailed metrics about the differences observed in the benchmarking if they had used 0.9 (or other intermediate value) as the threshold. For example, in the case of the supplementary table 2 (in vitro analysis), using a different cosine similarity threshold would result in SUITOR having a reduced precision/specificity compared to the other three tools, especially SigneR and SigProfilerExtractor. The selection of the cosine similarity threshold is critical considering that the average cosine similarity expected between two random nonnegative vectors simply by chance is 0.75 (doi: 10.1186/s12859-020-03772-3).
Response: This is a very good point. We followed this comment and provided additional results for the differences observed in the benchmarking between the thresholds 0.8 and 0.9.

For in vitro analysis, we added that “Next, we extracted de novo signature profiles and compared them with signature profiles reported in the original in vitro study. Indeed, de novo signatures extracted by SUITOR, SigProfilerExtractor, SignatureAnalyzer and signeR could be matched to 10, 5, 9, 6 of 11 in vitro true signatures respectively with the cosine similarity threshold 0.8 (sensitivity = 91%, 45%, 82%, 55%, Supplementary Table 2). The signature of 1,2-dimethylhydrazine (1,2-DMH) was detected by SUITOR and missed by the other methods. When cosine similarity threshold was increased to 0.9, the numbers of matched signatures were reduced to 7, 5, 7, 6 for SUITOR, SigProfilerExtractor, SignatureAnalyzer and signeR, respectively (sensitivity = 64%, 45%, 64%, 55%, Supplementary Table 2).” (Lines 203 to 211 on pages 9 and 10)

For In silico simulation studies of nine signatures, we commented that “The cosine similarities of five common signatures (SBS1, 2, 3, 5, 18) were all over 90% for 4 methods; the cosine similarities of SBS13 were slightly lower than 90% for SignatureAnalyzer and signeR and higher than 90% for the other two methods; as expected, the cosine similarities of two rare signatures (SBS8,41) were much lower than other signatures (except cosine similarities of SBS41 by SUITOR and SignatureAnalyzer). As a result, the increasement of cosine similarity threshold from 0.8 to 0.9 would impact the frequency of signatures SBS8, 13, 41 to be detected across 20 replicates but not other signatures (Supplementary Table 4).” (Lines 263 to 270 on page 12)

For In silico simulation studies of eight signatures with error mutation counts, we stated that “

Compared to the given signature profiles, cosine similarities of signatures detected by SUITOR were higher than 90% for SBS4, 6, 7a, 17b, 22, 26 and 39 and slightly lower than 90% for SBS9 (Supplementary Fig. 9b). Consequently, if the cosine similarity threshold is increased to 0.9, only the frequency of signature SBS9 to be detected across 20 replicates would be reduced for all methods (Supplementary Table 5), and SUITOR could still detect SBS9 in more replicates than other methods.” (Lines 282 to 288 on page 13)

2. For the real data analyses, the authors validate their results by indicating the matching of the extracted signatures to COSMIC reference signatures. This matching by itself is not an indication that the tool is identifying real signatures in the data. For example, in the analysis of the C. elegans dataset, the authors claimed to identify a signature closely matching SBS7c. This signature, as indicated, has a specific etiology (UV light exposure) and is usually found alongside SBS7a/b in skin tumors. Therefore, it seems unlikely that this signature is present in the original dataset by itself and could potentially represent a false positive signature. In addition, the authors should compare their results with the ones from the original C. elegans study (doi: 10.1038/s41467-020-15912-7).
Response: Following this comment, we compared de novo signatures detected by SUITOR with the ones from the original C. elegans study, for which the cosine similarities are much higher. In the manuscript, we stated that “SUITOR could detect five de novo SBS signatures. Compared to the signatures reported in the original C. elegans study, two de novo SBS signatures are highly similar to signatures of alkylating agents dimethyl sulfate (DMS, cosine similarity = 0.94, Supplementary Table C elegans) and ethyl methanesulfonate (EMS, cosine similarity = 1.00), respectively; and another two de novo SBS signatures are similar to signatures of DNA repair deficiencies due to genes SLX1 (cosine similarity = 0.89) and MLH1 (cosine similarity = 0.87), respectively.” (Line 396 to 402 on page 18)

3. Also regarding the C. elegans dataset, the authors should clarify if they have renormalized the COSMIC reference signatures to account for the differences in the trinucleotide proportions between human and C. elegans genomes. One example of the renormalization can be observed in the COSMIC website (<https://cancer.sanger.ac.uk/signatures/downloads/>), where the original GRCh37-based reference signatures were translated to different reference genomes and species, according to the different trinucleotide frequencies.
Response: Following the comment #2, we compared with signatures reported from the original *C. elegans* study instead. (Line 396 to 402 on page 18)

4. The authors should explain why they chose to include different noise levels generated from a uniform distribution, considering the data is an integer (counts). An expansion of the methods regarding the noise model selection would also be helpful.
Response: We clarified in the main text that the number generated from the uniform distribution would be rounded to the nearest integer. (Lines 274 to 276 on page 13) Moreover, we expended the methods section regarding the noise model selection,commenting that “In addition, we added error mutation counts for each mutation type and each tumor caused by possible sequencing and/or calling errors; we chose a relatively simple noise model to imitate error mutation counts not specific to a subset of mutation types. Specifically, error mutation counts were generated independently and identically from a uniform distribution for each mutation type with the range [0, a$\times$b] and were rounded to the nearest integer, where the error level a equates to 0 (i.e., no error mutation counts), 0.4, 0.8 and 1.2, and b is the average mutation count of each tumor (i.e., b being the column average of the matrix $\mathbf{V}^{\mathbf{0}}$). Hence, the error mutation counts were proportional to counts of true mutation counts with varying error levels.” (Lines 639 to 647 on page 29)

5. COSMIC Mutational Signatures v3.2 includes 78 single base substitution signatures and not 49, since several artifact-related signatures are also included in the reference set.
Response: we have corrected it. (Line 54 to 55 on page 3)

6. The results of the application of SUITOR to hypermutated tumors, indels, and the C. elegans study should be included in the results section apart from the discussion section. For the analysis of hypermutators and indel signatures, it should be indicated which datasets were used and how many samples were included per group. For indels and the C. elegans study, a supplementary table with the cosine similarities similar to the ones provided for the other datasets is missing.
Response: Following this comment, we moved the results of the application of SUITOR to hypermutated tumors, indels, and the *C. elegans* study to the Results section; datasets and sample sizes of hypermutators and indel signatures were indicated in the manuscript; we added cosine similarities table for indels and the *C. elegans*. (Lines 381 to 413 on pages 18 and 19)

7. For SignatureAnalyzer, it should be specified if the CPU or the GPU version was used.
Response: The CPU version was used, and we clarified it in the revised manuscript. (Line 708 on page 32)

8. The output W and H matrices are missing for the newly included datasets of PCAWG Colorectal, PCAWG Esophageal, PCAWG Head and Neck, ovarian adenocarcinoma for indel analysis, and C. elegans analysis. Also, to benefit reproducibility, the mutational matrices used as input for all benchmarking and real data analyses should be included in the supplementary dataset.
Response: We added the output W and H matrices of PCAWG Colorectal, PCAWG Esophageal, PCAWG Head and Neck, ovarian adenocarcinoma for indel analysis, and C. elegans analysis; in addition, the mutational matrices were included in the supplementary dataset as well.

9. Typos: spacing between equal signs not consistent (e.g., lines 389-391); C. elegans should be written in italics (lines 394-397); *caner (line 407). Please check for typos in the user guide too (e.g., *SUTIOR, section 4.2).

Response: These typos were corrected.
